# The glycine-rich domain of GRP7 plays a crucial role in binding long RNAs and facilitating phase separation

**DOI:** 10.1038/s41598-024-66955-5

**Published:** 2024-07-11

**Authors:** Kim Lara Lühmann, Silja Seemann, Nina Martinek, Steffen Ostendorp, Julia Kehr

**Affiliations:** Department of Biology, Molecular Plant Genetics, Institute of Plant Science and Microbiology, Universität Hamburg, Hamburg, Germany

**Keywords:** RNA-binding proteins, Plant sciences

## Abstract

Microscale thermophoresis (MST) is a well-established method to quantify protein-RNA interactions. In this study, we employed MST to analyze the RNA binding properties of glycine-rich RNA binding protein 7 (GRP7), which is known to have multiple biological functions related to its ability to bind different types of RNA. However, the exact mechanism of GRP7’s RNA binding is not fully understood. While the RNA-recognition motif of GRP7 is known to be involved in RNA binding, the glycine-rich region (known as arginine-glycine-glycine-domain or RGG-domain) also influences this interaction. To investigate to which extend the RGG-domain of GRP7 is involved in RNA binding, mutation studies on putative RNA interacting or modulating sites were performed. In addition to MST experiments, we examined liquid–liquid phase separation of GRP7 and its mutants, both with and without RNA. Furthermore, we systemically investigated factors that might affect RNA binding selectivity of GRP7 by testing RNAs of different sizes, structures, and modifications. Consequently, our study revealed that GRP7 exhibits a high affinity for a variety of RNAs, indicating a lack of pronounced selectivity. Moreover, we established that the RGG-domain plays a crucial role in binding longer RNAs and promoting phase separation.

## Introduction

RNA binding proteins (RBP) participate in a variety of different cellular functions like splicing, translation, RNA transport, and stress response. Some RBPs contain highly conserved RNA binding domains such as the K homology domain^1^, RNA recognition motif (RRM)^2,3^ or ZINC-finger domain^4^. The RRM is a well-folded RNA-binding domain with a size around 80 amino acids (aa) and consists of two conserved sequence motifs, RNP1 and RNP2, which are interacting preferably with ssRNA^2^. Besides RNA binding domains with conserved structures, a huge number of RBPs contain intrinsically disordered regions (IDR) like the RGG-domain which also has RNA binding function^5,6^. Some members of the hnRNP-family, like hnRNP-A1 and hnRNP-A2, contain an RRM as well as an IDR^7^. Likewise, the plant glycine-rich RNA binding protein 7 (GRP7) is a 17 kDa small protein containing an RRM and a glycine-rich IDR^8^. Its glycine-rich region (RGG-domain) contains besides glycine tyrosine, serine and arginine. The high percentage of glycines within the RGG provides a high order of flexibility while other amino acids like arginine and tyrosine interspersed between stretches of glycine have space for intra- and intermolecular interactions and are easily modified^9,10^. Often, IDR containing proteins undergo phase separation which is enabled by the high flexibility and the open access to side chains of other amino acids within this region^10^. The RRM is considered to play a major role in RNA binding of GRP7^11^ while the RGG-domain was shown to interact with transportin1 which facilitates the movement of GRP7 between nucleus and cytosol^12^. Furthermore, the RGG-domain can influence GRP7s RNA-binding affinity^11^. Moreover, GRP7 might be involved in RNA (long-distance) transport, since the RGG-domain promotes cell-to-cell movement of GRP7^13^ and GRP7 was found in phloem sap of *Brassica napus* (*B.* *napus*)^14^.

Multiple biological functions of GRP7 like alternative splicing, cold resistance and pathogen resistance, are connected to its ability to bind RNA. GRP7 is a circadian clock slave oscillator, regulating its own and other transcripts by alternative splicing of pre-mRNA^15–17^. Besides this, GRP7 was reported to facilitate mRNA export from the nucleus during cold stress^18^. Through the interaction with small, single stranded siRNA, the small RNA binding protein 1 from tobacco, a homologue of GRP7, was proposed to slow down the systemic spread of Turnip mosaic virus (TuMV)^13^. Thus, GRP7 is a versatile protein with many functions capable of binding small as well as large RNA. Nevertheless, previously conducted RNA interaction studies mainly focused on small oligonucleotide probes of its own pre-mRNA and did not test complete mRNAs^11,16,19–21^.

With microscale thermophoresis (MST), biomolecular interactions can be investigated by a micro-temperature gradient induced fluorescence change (thermophoresis)^22^. Besides MST, other methods like electromobility shift assays (EMSA), isothermal titration calorimetry (ITC) and surface plasmon resonance spectroscopy (SPR) are used for the determination of dissociation constants. All of these methods have advantages and disadvantages. While EMSA is cheap and can be used in any laboratory without the requirement of using specialized equipment, it only allows a semi-quantitative determination of protein-RNA interactions^23^. ITC on the other hand can be used for quantitative assessment of protein-RNA interaction and none of the interaction partners has to be labeled, but it requires a comparably high quantity of sample as the binding heat needs to be measurable^24,25^. Like ITC, SPR is a versatile method for quantitative analysis of protein-RNA interactions, but one interaction partner has to be immobilized to a thin metal surface like gold, thus the interaction might be influenced due to the change of dynamics of the immobilized interaction partner^26^. Therefore, MST was chosen, since it enables quantitative analysis of interaction in solution and does only require small amounts of sample^22^. For the temperature gradient inducing the thermophoresis, an infrared laser is used to heat the sample within a capillary at a precise spot while another laser is used to excite the fluorophore attached to one of the interaction partners. By measuring the fluorescence in the heated spot, changes in fluorescence during thermophoresis can be measured^27^. The thermophoresis of a molecule is influenced by size, charge, conformation and interaction with the surrounding solvent. Since all of these factors are influenced by the interaction with another molecule^27^, the detected change in fluorescence during thermophoresis is modified by intermolecular interactions of the fluorescently labeled molecule with other molecules. By preparing a titration series with the unlabeled molecule and the addition of a constant concentration of the labeled molecule, MST can be used to determine the dissociation constant of an interaction, like a protein-RNA interaction in solution^22^. For example, MST was used to determine the dissociation constant of a poly-A binding protein (PABP) with differently modified poly-A sequences^28^ or of SARS-CoV-2 nsp13 protein with G-quadruplex structures in SARS-CoV-2-RNA^29^.

To further understand the binding properties of GRP7 and mutants towards RNAs differing in length, sequence, modification and structure, MST was used to determine the binding affinities. Additionally, the influence of truncation of GRP7 and mutations within the RGG-domain on its phase separation behavior with and without RNA was studied. Our results suggest that GRP7 is capable to bind a variety of RNAs with high affinity and underline the importance of the RGG-domain of GRP7 for RNA binding and phase separation.

## Results

### Binding affinity of AtGRP7 towards different RNAs

**Figure 1:**
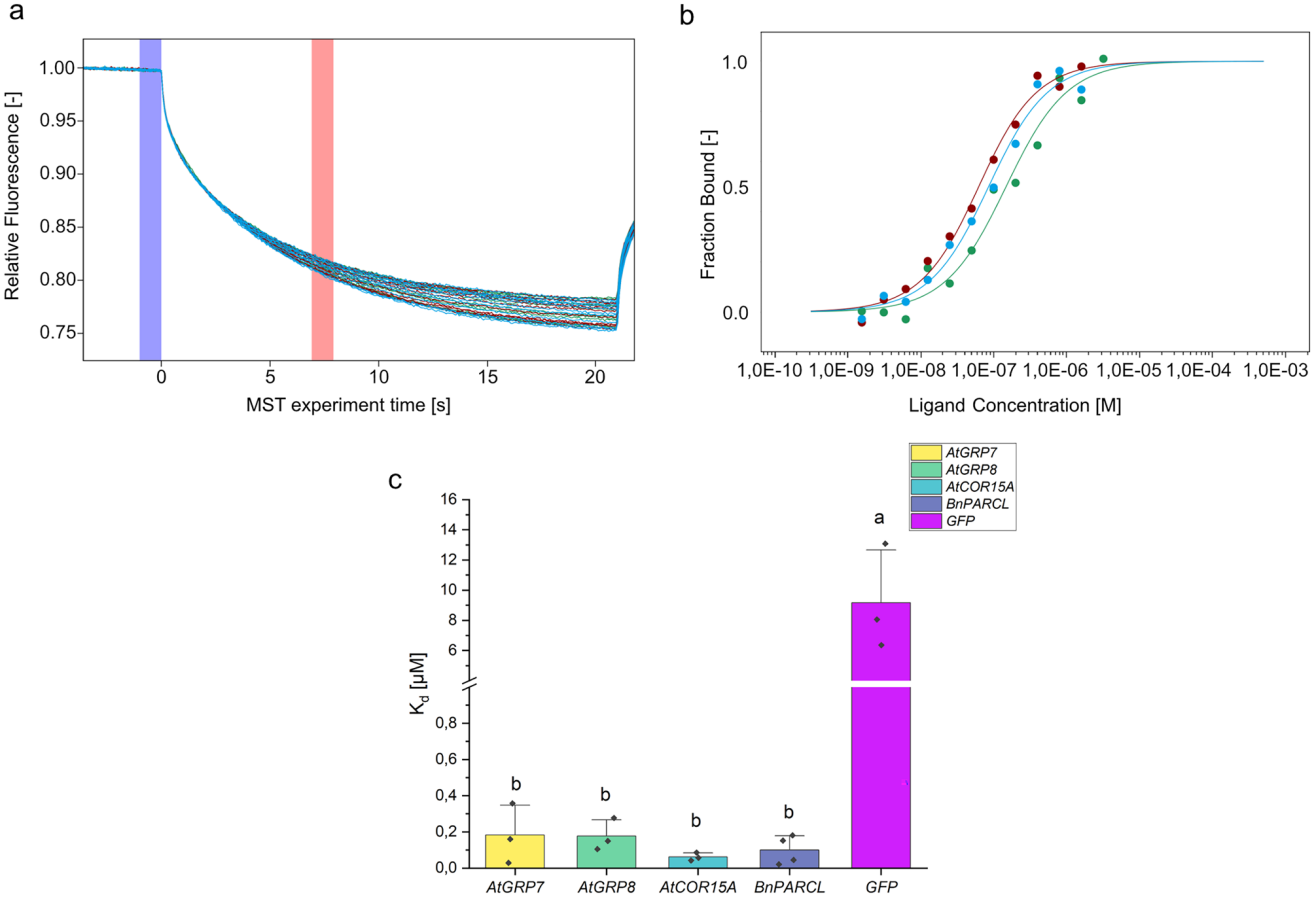
Analysis of the RNA binding of AtGRP7 via microscale thermophoresis (MST). (**a**) Thermophoresis traces of three individual measurements (1–3, displayed in blue (1), green (2) and red (3)) with a titration series of 16 of AtGRP7 and a constant concentration of *AtCOR15A*. The x-axis displays the time (s) of the thermophoresis experiment, 0 s as start of the thermophoresis. The y-axis displays the relative fluorescence measured in the heated area. The blue area marks F_cold_ while the red area marks F_hot_, both used to calculate the binding curve. (**b**) Binding curves of three individual measurements (1–3, displayed in blue (1), green (2) and red (3)) of AtGRP7 and *AtCOR15A*. The x-axis displays the Ligand concentration (M, logarithmic). The y-axis displays the fraction bound, from 0 (no RNAs bound) to 1 (all RNAs bound). (**c**) K_d_s of different RNAs bound by AtGRP7 shown in a bar graph. The x-axis displays the RNA measured and the y-axis the K_d_ in µM with a cut between 1 and 4 µM.

GRP7 is known to interact with different kinds of RNAs, like small RNAs^13^ and pre-mRNAs^30^. iCLIP data combined with RIP-seq data showed the enrichment of 452 mRNAs by GRP7^31^. From these RNAs, three RNAs were chosen to investigate the interaction with GRP7 via MST including the *AtGRP7* transcript, *AtGRP8* and *AtCOR15A*. For all RNAs, the CDS was amplified and in vitro transcribed, including Cy5-labeled UTP. To investigate binding affinity of AtGRP7 towards RNAs that are no natural targets, *BnPARCL* and *GFP* were used. MST data was evaluated at 6.92 s on time as seen as red bar in thermophoresis graphs (Fig. 1a) for all mRNAs tested. From the thermophoresis graphs, a binding curve was calculated (Fig. 1b). AtGRP7 showed a low dissociation constant (K_d_) for all three tested RNAs in a range of 0.062 µM ± 0.022 µM (*AtCOR15A*) to 0.182 µM ± 0.165 µM (*AtGRP7*) (Fig. 1c, Supplementary Tab. S5).

Due to GRP7s possible function in long-distance RNA transport through the phloem, we were interested which phloem RNAs can be bound by GRP7. For sampling higher amounts of phloem sap, a close relative of *A. thaliana*, *Brassica napus*, was chosen to sample phloem sap and extract RNA. Therefore, also a homologue of AtGRP7 in *B. napus*, BnGRP7 (BnaC08g49360D), was used in following tests to ensure RNA binding was not influenced by using the protein of one plant species and RNA of another. To test, which phloem RNAs are bound to BnGRP7, the protein was coupled on Sepharose™ beads, incubated with total phloem RNA of *B. napus*, bound RNA was eluted and analyzed with bioanalyzer (Supplementary Figs. S1, S2). The samples were then further subjected to library preparation of poly-A enriched and small RNA libraries and subsequent Illumina sequencing, all performed by Novogene. The results were compared to Illumina sequencing data of total phloem RNA prior incubation with BnGRP7-beads to determine a potential selectivity of BnGRP7 for certain RNAs. However, within eluted RNA from BnGRP7 only a few enriched RNAs were identified with a low log2fold change (< 2) and over 40,000 mRNAs were bound (Supplementary Fig. S3, Supplementary Tab. S1 and S2). For small RNAs, no enrichment was detected in the elution fractions compared to input RNAs (Supplementary Fig. S3, Supplementary Tab. S3). Therefore, BnGRP7 did not show selectivity for certain RNAs, but was able to bind a broad range of small and large RNAs (Supplementary Fig. S4).

### Influence of RNA length, UTRs and ss/dsRNA on GRP7 binding affinity

**Figure 2:**
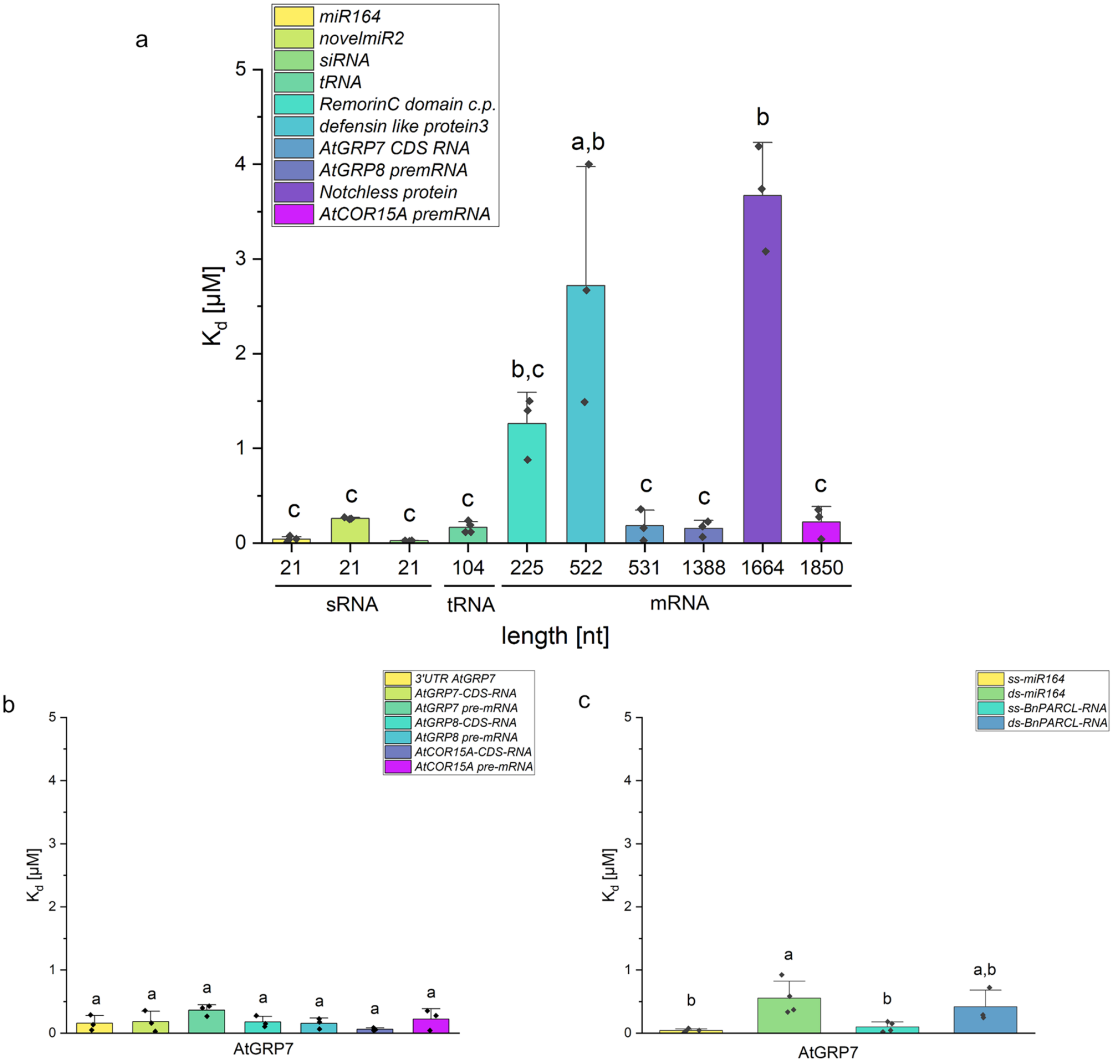
Comparison of dissociation constants (K_d_s) of AtGRP7 for RNAs varying in length, with and without UTRs and Introns, as well as single and double stranded RNA. (**a**) K_d_s of AtGRP7 for RNAs varying in length displayed in a bar graph. The length of RNAs is shown on the X-axis, the Y-axis shows the K_d_ in µM. K_d_s were compared by one-way ANOVA and Tukeys test (p = 0.01). Similar letters indicate no significant difference. (**b**) Comparison of K_d_s of AtGRP7 for RNAs with and without UTRs and Introns. Y-axis shows the K_d_ in µM. K_d_s were compared by by one-way ANOVA and Tukeys test (p = 0.05). Same letters indicate no significant difference. (**c**) Comparison of K_d_s of AtGRP7 for ssRNA and dsRNA. Y-axis shows the K_d_ in µM. K_d_s were compared by one-way ANOVA and Tukeys test (p = 0.05). Same letters indicate no significant difference.

Besides interacting with mRNAs, GRP7 was shown to interact with siRNAs during virus infection^13^. To get a deeper understanding of GRP7s binding selectivity for different kinds of RNAs, MST was performed with small RNAs, tRNA and mRNAs of different lengths. Selected miRNAs, *Remorin C-domain*, *defensin like protein 3* and *Notchless protein* RNA were found in the elution fraction of phloem RNAs bound by BnGRP7 (Supplementary Tab. S1, S2), while *AtGRP7*, *AtGRP8* and *AtCOR15A* were chosen because they are known to be bound by GRP7^31^. The results showed some significant differences in binding affinities of AtGRP7 for long and short RNAs. However, the difference in binding affinity towards short and long RNA was not correlated to the length of RNA. GRP7 showed a dissociation constant (K_d_) of 0.15 µM towards the over 1800 nt long *AtCOR15A* pre-mRNA while it had a significant higher K_d_ of 2.72 µM towards the 522 nt long RNA of *defensin like protein 3* (Fig. 2, Supplementary Tab. S5). To investigate if GRP7 prefers specific RNA structures, in silico structure predictions were performed. The results showed that some of the RNAs, like *GFP* and *Notchless protein*, were highly structured and bound with lower affinity by GRP7. Other RNAs, like *AtGRP7 pre-mRNA* and *AtCOR15A pre-mRNA*, were less structured and bound by GRP7 with higher affinity (Supplementary Fig. S7), indicating a tendency that GRP7 binds less structured RNAs better than RNAs with more structure. By testing a G-quadruplex RNA sequence, which was previously used to identify the G-quadruplex binding behavior of fragile X mental retardation protein (FMRP)^32^, this tendency was further substantiated. As LiCl resolves the structure of G-quadruplex RNAs, MST was performed with buffer containing 150 mM LiCl instead of NaCl. LiCl increased the affinity of GRP7 towards *Sc1* RNA significantly (Supplementary Fig. S8), confirming a preference of GRP7 for less structured RNA. Another factor possibly influencing the binding affinity of AtGRP7 is the presence of specific sequences. As GRP7 is known to bind its own pre-mRNA within the 3’UTR according to iCLIP and RIP-seq data^31^, the binding affinities of GRP7 for RNAs with and without their native UTRs were tested. The K_d_s of AtGRP7 for RNAs with and without UTRs and introns were compared as well as the binding affinity of AtGRP7 towards a 32 nt long fragment of its own 3’UTR which was found to be a binding spot of AtGRP7 and has already been tested with EMSA^11^. No significant differences in binding affinity were found between UTR and intron-containing RNAs and only CDS RNAs (Fig. 2b, Supplementary Fig. S12). GRP7 also showed many binding spots within exons^31^, what can explain why the RNAs without UTRs were bound. For both, UTRs and exons, a similar UC-rich sequence was bound by GRP7^31^. The results confirm the observation that GRP7 has a broad RNA binding capability.

Furthermore, GRP7 was described to show a binding preference for ssRNA^19^, the RRM preferably interacting with single stranded RNAs^2^. To verify this preference, the binding affinities of AtGRP7 for ssRNA and dsRNA were compared. However, only ss-miR164 was bound by GRP7 with a significantly higher affinity than ds-miRNA. For longer RNA, in this case the 455 nt long *BnPARCL*-RNA, no significantly different affinity for dsRNA was observed, but GRP7 showed a tendency for higher affinity towards ss-*BnPARCL*-RNA, indicating a possible preference of AtGRP7 to bind ssRNA.

### Methylation of RNA does not influence the binding affinity of GRP7

**Figure 3:**
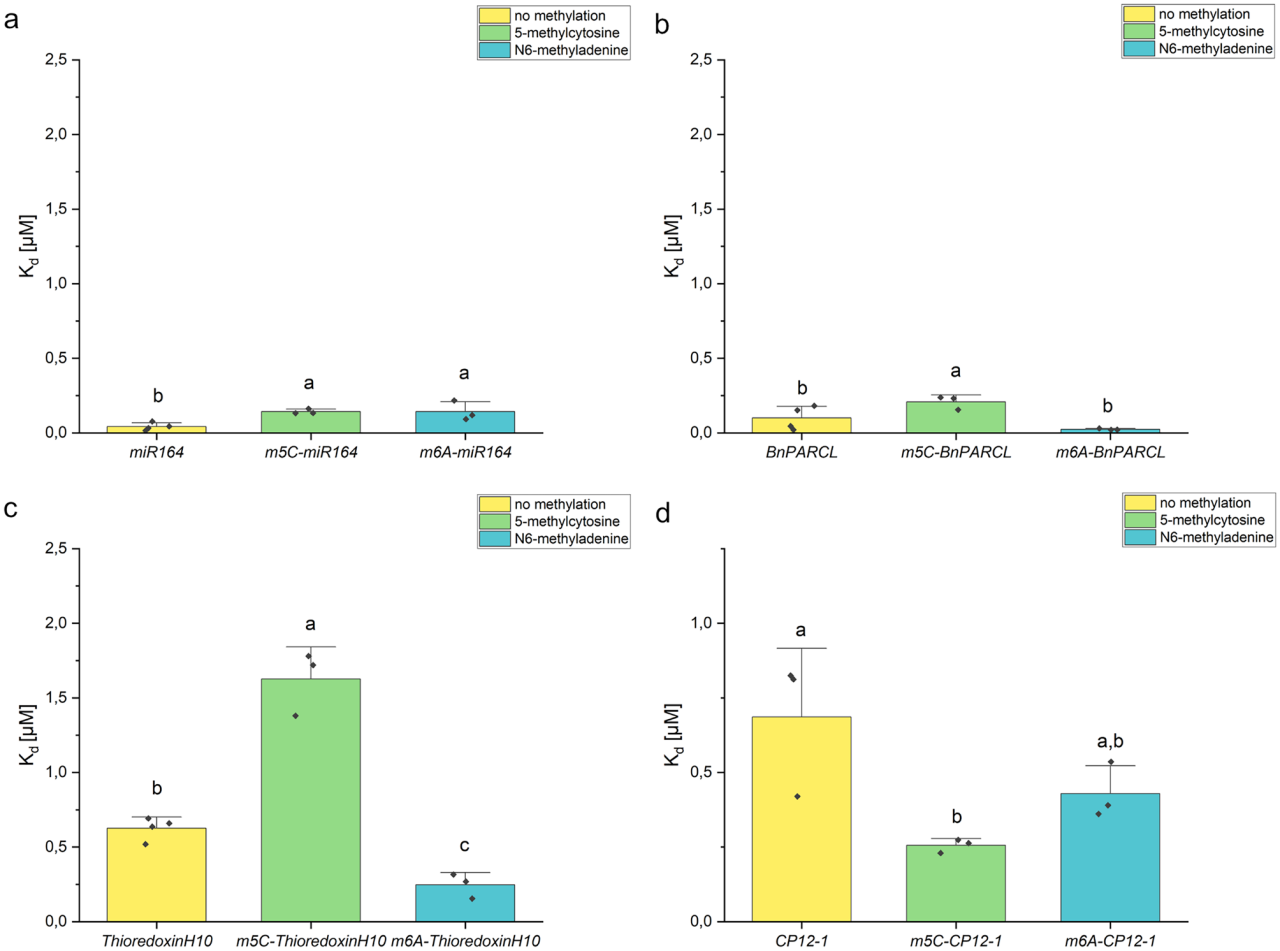
Dissociation constants (K_d_s) of AtGRP7 for methylated RNA. (**a**–**d**) display the binding affinities of AtGRP7 towards four different RNAs with and without methylation. Y-axis: K_d_ in µM, X-axis: different RNA with different methylations. The binding affinity of AtGRP7 for methylated and unmethylated RNA was compared with a one-way ANOVA and a Tukeys test for p = 0.05. Shared letters indicate no significant difference in binding affinity.

One frequent post-transcriptional modification of RNAs is the addition of methyl groups. In plants, methylation of adenosine (m6A) is the most abundant mRNA modification which is important for growth and development^33^. Often, m6A methylations are present within the 3’UTR of RNA^34^ which is also a preferred binding spot of AtGRP7^31^ and GRP7 was previously identified as a putative m6A binder^35^. Therefore, such a methylation might alter the binding selectivity of GRP7 towards RNA. Another common RNA modification is the methylation of cytosine (m5C). This methylation was shown to promote long-distance mobility of some mRNAs like *TCTP*^36^, thus GRP7 might show a preference for binding m5C methylated RNA, since GRP7 was found in the phloem^14^. *THIOREDOXINH10* and *CP12-1* RNAs were found in phloem samples and *CP12-1* was also found in the fraction of AtGRP7 bound phloem RNAs (Supplementary Tab. S1 and S2). To test if AtGRP7 has a preference to bind methylated RNAs, RNAs containing m5C or m6A methylations were in vitro transcribed and MST was used to measure the binding affinity of GRP7. AtGRP7 did not show a consistent preference for unmethylated, m5C or m6A methylated RNAs. For two RNAs, *BnPARCL* and *THIOREDOXINH10*, the binding affinity of GRP7 for the m6A methylated RNA was higher than for unmethylated RNA, but only for one of them *m6A-THIOREDOXINH10* it was significant (p = 0.05). On the other hand, for *m5C-miR164*, *m5C-BnPARCL* and *m5C-THIOREDOXINH10* GRP7 showed a significantly lower (p = 0.05) binding affinity than for the respective non-methylated RNAs. These results indicate that AtGRP7 has no preference for methylated RNAs (Fig. 3, Supplementary Tab. S4).

Since all methylated RNAs tested were RNAs from *B. napus*, the same test was performed with BnGRP7. For two of the RNAs, *miR164* and *BnPARCL-RNA*, the binding affinity of BnGRP7 for non-methylated RNA was significantly higher (p = 0.05) than for the m5C and m6A methylated RNA. For the other two RNAs, *THIOREDOXINH10* and *CP12-1-RNA*, no significant difference (p = 0.05) between the binding affinities of BnGRP7 for methylated and unmethylated RNA was observed (Supplementary Fig. S6, Supplementary Tab. S6). These results were coherent with the results for AtGRP7.

### The *glycine*-rich domain is important for a high binding affinity of GRP7

**Figure 4:**
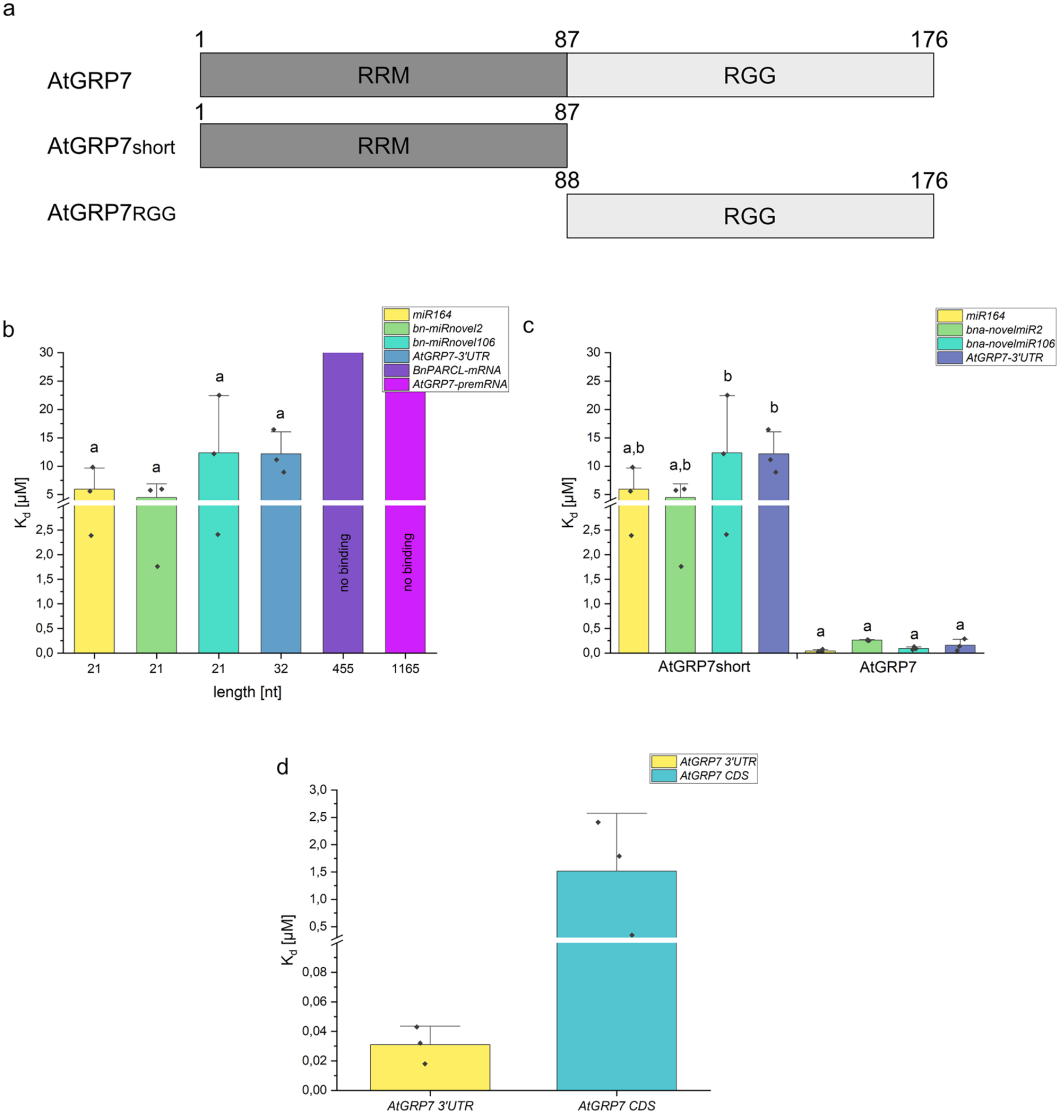
Dissociation constants of AtGRP7^short^ for small and long RNAs. (**a**) AtGRP7 consist of two domains, the RNA-recognition motif (RRM) from 1 to 87 aa and the glycine-rich region (88–176 aa), while the truncated AtGRP7^short^ only consist of the RRM. AtGRP7^RGG^ consist of the RGG-domain. (**b**) Binding affinity of AtGRP7short for small and long RNAs, shown in a bar graph. The K_d_ of AtGRP7^short^ towards the different RNAs was compared by a one-way ANOVA with a Tukeys test (p = 0.05). (**c**) Comparison of AtGRP7 and AtGRP7short RNA binding affinities towards small RNAs, shown in a bar graph. The Y-axis displays the K_d_ in µM cut between 3 and 4 µM. The binding affinity of AtGRP7 and AtGRP7^short^ towards the different RNA was compared by a one-way ANOVA with a Tukeys test (p = 0.05). Shared letters indicate no significant different K_d_. (**d**) Binding affinity of the eYFP-tagged AtGRP7^RGG^ for *AtGRP7 3′UTR* and *AtGRP7 CDS* RNA displayed in a bar graph. The y-axis resembles the K_d_ in µM and is cut between 0.1 and 0.3 µM.

The RRM is a known RNA-recognition site and a common domain for RNA binding proteins. It is known that RNA interacts with two specific areas, RNP1 and RNP2 of the RRM, both located in the beta-sheets of the RRM^2^. For GRP7, the RRM is crucial for its RNA binding capability, since mutation of alanine 49 (located in RNP1) leads to a lower RNA binding affinity^15^. However, not only the RRM interacts with the RNA, the RGG-domain of AtGRP7 can interact with the RRM^37^ and a removal lead to a decrease in RNA binding ability, thereby indicating a supportive role of the RGG-domain in RNA binding^11^. To confirm this with MST, a truncated version of AtGRP7 (AtGRP7^short^) only containing the RRM was tested for its binding affinity towards RNAs of different length. The truncation was introduced directly after the RRM amino acid 87 (Fig. 4a). AtGRP7^short^ showed dissociation constants in the low micromolar range for small RNAs (Fig. 4b). Comparing the binding affinity of AtGRP7 and AtGRP7^short^ for small RNAs, the RNA binding affinity of AtGRP7^short^ was significantly lower than the binding affinity of the full-length protein (Fig. 4c, Supplementary Tab. S5 & S7). For longer RNAs, no binding was detected. To confirm that the RGG-domain of AtGRP7 alone can bind RNAs, AtGRP7^RGG^ was purified fused to an N-terminal eYFP. Removal of this eYFP resulted in the rapid formation of condensates and aggregates that precluded measurements. However, the eYFP-tagged AtGRP7^RGG^ bound *AtGRP7 3’UTR* as well as *AtGRP7 CDS* RNA (Fig. 4d) with high and medium affinities between 0.031 and 2.1 µM, while eYFP did not bind RNA (Supplementary Fig. S9). Thus, the RGG-domain contributes to the ability of GRP7 to bind longer RNAs and can even bind RNAs alone, but is not crucial for sRNA binding.

**Figure 5:**
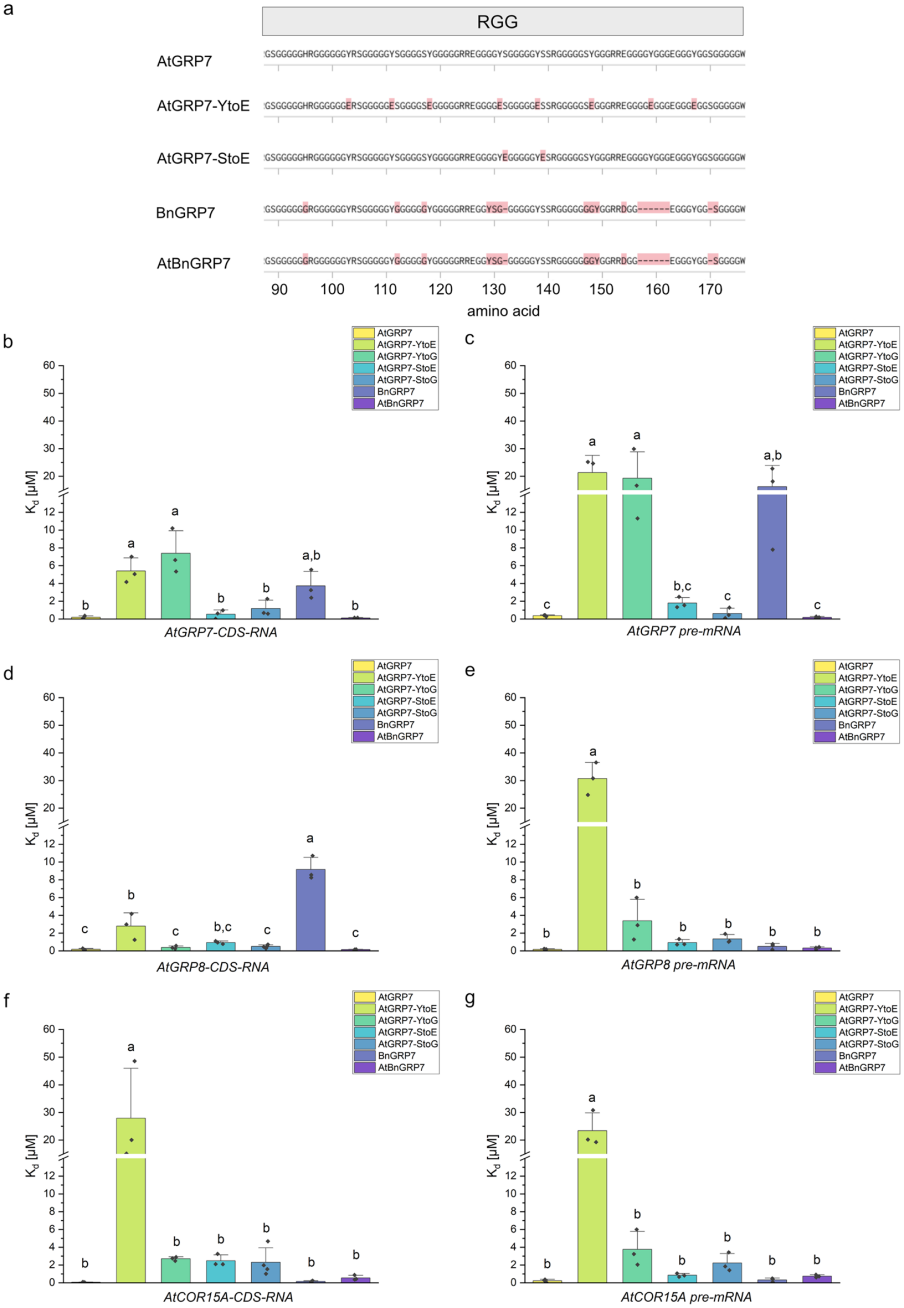
Mutations and differences in the glycine-rich region of GRP7 and their effect on the dissociation constants (K_d_s) for different RNAs. (**a**) The RGG-domain of AtGRP7, starting at amino acid 88, in comparison with the RGG-domain of two mutants, AtGRP7^YtoE^ and AtGRP7^StoE^ as well as to BnGRP7 and the hybrid protein AtBnGRP7. Differences are highlighted in red. (**b**–**g**) Binding affinity of AtGRP7, AtGRP7^YtoE^, AtGRP7^YtoG^, AtGRP7^StoE^, AtGRP7^StoG^, BnGRP7 and AtBnGRP7 for *AtGRP7* (**b**), *AtGRP7* pre-mRNA (**c**) *AtGRP8* (**d**), *AtGRP8 pre-mRNA* (**e**), *AtCOR15A* (**f**) and *AtCOR15A pre-mRNA* (**g**). Y-axis: K_d_ in µM, cut between 12 and 13 µM. The binding affinities of AtGRP7 and different versions of GRP7 towards different RNAs were compared with one-way ANOVA and a Tukeys test for p = 0.05. Shared letters indicate no significant difference in binding affinity.

To further investigate the involvement of the RGG-domain in RNA binding of GRP7, mutations were introduced. The domain contains eight tyrosines and tyrosine plays a major role in protein-nucleic acid interactions through pi-pi stacking and hydrogen-bond formation with nucleotide bases^10^. Thus, it is likely that the RGG-domain is interacting with RNA through these tyrosines. Some of the tyrosines and serines of GRP7 were shown to be phosphorylated by FERONIA (FER)^38^. To test if the modifications within the RGG-domain have an effect on RNA binding, all tyrosines in the RGG-domain were mutated to glutamic acid (E) to introduce a negative charge similar to the effect of a phosphorylation. As a control, all eight tyrosines were mutated to glycines (G). Since only serine 134 and serine 139 phosphorylation sites showed phosphorylation in planta^38^, both serines were mutated to either glutamic acid or glycine as control (Fig. 5a). Additionally, BnGRP7 was investigated. BnGRP7 shows a high similarity towards AtGRP7, but has some differences within the RGG-domain (Fig. 5a). The RGG-domain of BnGRP7 is shorter and contains one tyrosine less than AtGRP7. To test whether differences in binding affinities towards certain RNAs are due to the differences in their RGG-domain, the mutant AtBnGRP7 was prepared, containing the RRM-domain of AtGRP7 and the RGG-domain of BnGRP7. To ensure that the mutations and the removal of the RGG-domain did not cause significant structural changes, the structures of AtGRP7, BnGRP7 and AtGRP7 mutants were predicted with AlphaFold 2 (v2.3.1)^39^. For all predictions, the zero-ranked structures were chosen and aligned with the structure alignment tool of RCSB PDB using the jFATCAT-flexible algorithm (v2.0)^40,41^. The alignment showed that the structures of all mutants were similar to the AtGRP7 wildtype protein (Supplementary Fig. S10). As the mutations targeted the RGG-domain, which is an intrinsically disordered region with no predicted structure, the overall structure of the protein was not affected by the mutations nor the truncation.

To investigate the RNA binding capability of GRP7 and the mutants, three different mRNAs were tested, *AtGRP7*, *AtGRP8* and *AtCOR15A* (Fig. 5, Supplementary Tab. S4, S5, S6, S7, S8, S9, S10, S11 and S12). All of these RNAs are known to interact with GRP7, whereby their splicing is affected^31^.

The binding affinities of AtGRP7^YtoE^ mutant were significantly lower for all of the tested RNAs compared to AtGRP7 (Fig. 5). Interestingly, when testing the binding affinity of AtGRP7^YtoE^ for a smaller RNA like the 32 nt long part of *AtGRP7-3’UTR*, the binding affinity was still significantly lower compared to AtGRP7, but it was not significantly lower comparing the affinity of both proteins towards a miRNA (Supplementary Fig. S11). Thus, the mutation of Y to E reduced the binding affinity of AtGRP7 significantly for longer RNAs, but not for smaller RNAs. Nevertheless, AtGRP7^YtoG^ only exhibited significantly different RNA binding affinities compared to AtGRP7 for *AtGRP7* and *AtGRP7 pre-mRNA*, but not for other RNAs. This indicates, that the introduction of a negative charge within the RGG-domain has a higher influence on RNA binding behavior than the exchange of tyrosine.

The dissociation constants of AtGRP7 and BnGRP7 were, for most of the RNAs, not significantly different. Nevertheless, BnGRP7 showed higher dissociation constants for some of the RNAs, thus a low binding affinity than AtGRP7. For *AtGRP8-CDS* RNA this difference was significant (Fig. 5d). However, this difference in binding affinity was not observed for AtBnGRP7. To make sure that this difference in binding affinity between AtGRP7 and BnGRP7 was not due to the usage of RNAs originating from *A. thaliana*, also *GRP7* RNAs from *B. napus* were tested. Here, BnGRP7 showed higher K_d_s than AtGRP7, and thereby a lower affinity towards its own RNA, while AtBnGRP7 had similar K_d_s compared to AtGRP7 (Supplementary Fig. S5), suggesting the binding affinity for specific RNAs relies on both, RRM- and RGG-domain.

As GRP7 is known to bind RNAs in their UTRs, binding affinity of AtGRP7, AtGRP7^StoE^ and AtGRP7^YtoE^ for CDS RNAs and pre-mRNAs were compared (Fig. 2b, Supplementary Fig. S12). Here, no significant difference in binding affinity between CDS RNA and pre-mRNA was observed.

### Liquid–liquid phase separation of AtGRP7 and mutants

**Figure 6:**
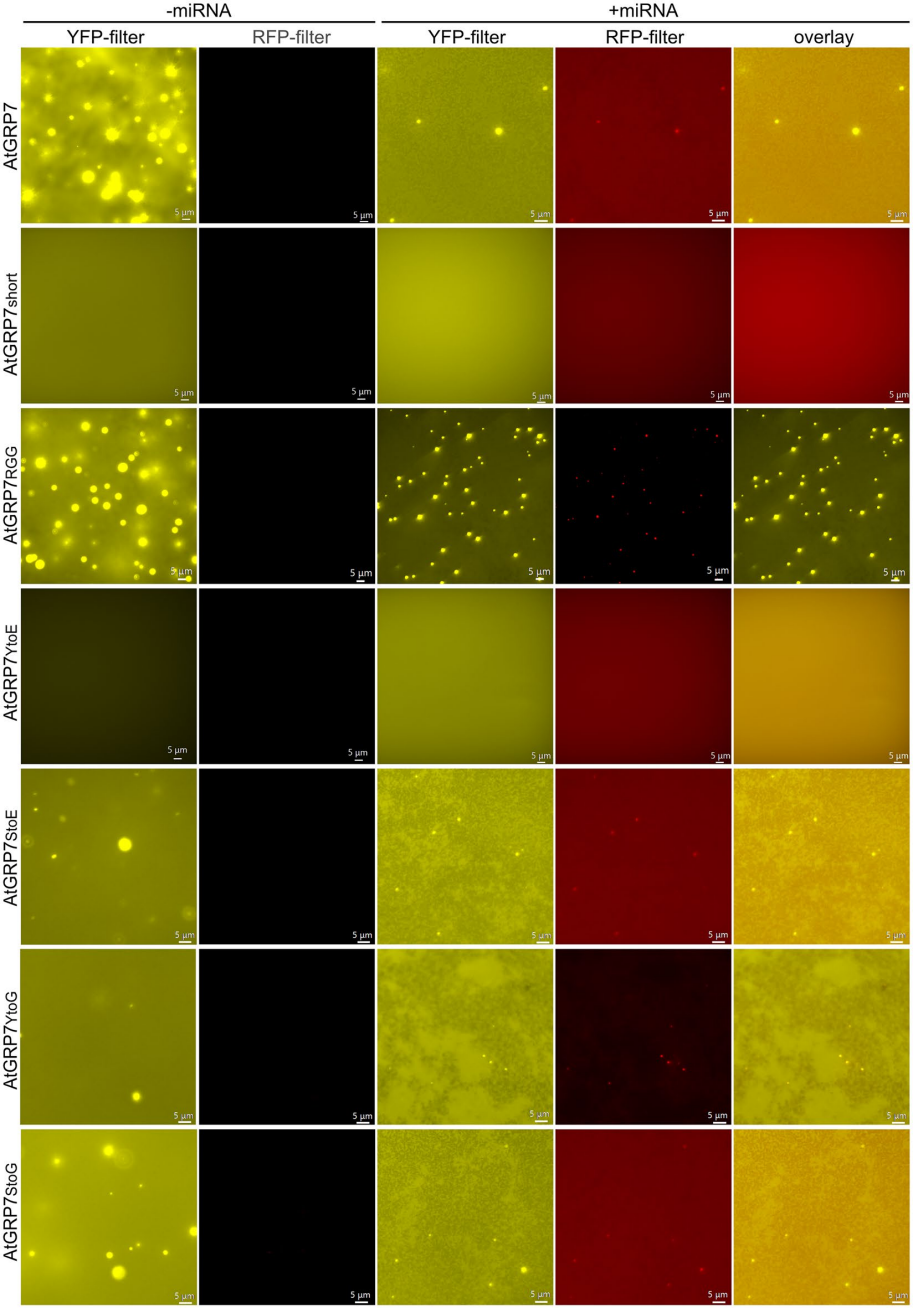
Liquid–liquid phase separation of AtGRP7 and mutants. 10 µM of eYFP-tagged AtGRP7, AtGRP7^short^, AtGRP7^RGG^, AtGRP7^YtoE^, AtGRP7^StoE^, AtGRP7^YtoG^ and AtGRP7^StoG^ and their phase separation behavior with and without 0.5 µM miRNA164. The white bar represents 5 µm.

Many proteins harboring an intrinsically disordered region (IDR) are able to undergo phase separation like hnRNP and FUS^42,43^. The glycine-rich region of AtGRP7 is one of those IDRs and is expected to perform liquid–liquid phase separation (LLPS), since first experiments showed the formation of liquid droplets^44^. To test if the glycine-rich region facilitates phase separation, AtGRP7 and two truncated version of AtGRP7 only containing the RRM (AtGRP7^short^) or only containing the RGG-domain (AtGRP7^RGG^) were tagged with eYFP on their N-terminus and tested for phase separation behavior. Furthermore, phosphorylation of tyrosine is known to modulate phase separation for hnRNPA2 and FUS^45^. It was shown that AtGRP7 can be phosphorylated by FERONIA (FER)^38^. In vitro, the phosphorylation of two tyrosines (Y^111^ and Y^138^) and four serines (S^112^, S^132^, S^139^ and S^140^) was confirmed^38^. To test if phosphorylation influences phase separation of AtGRP7, the two phosphomimic-mutants AtGRP7^YtoE^ and AtGRP7^StoE^ were used as well as the control mutants AtGRP7^YtoG^ and AtGRP7^StoG^. AtGRP7 showed phase separation at concentrations of 1 µM, 5 µM and 10 µM (Supplementary Fig. S13). AtGRP7^short^ barely showed phase separation, the same was true for AtGRP7^YtoE^(Fig. 6). AtGRP7^RGG^, AtGRP7^StoE^, AtGRP7^YtoG^ and AtGRP7^StoG^ showed phase separation (Fig. 6). This suggested that the RGG-domain is essential for AtGRP7 phase separation behavior. It is likely that the phosphorylation of tyrosines leads to a reduced condensate formation, since the mutant AtGRP7^YtoE^ rarely formed condensates. A comparable mutation in a protein with a comparable IDR, AtPARCL, showed similar results^46^. The Y to E mutant as well as the in vitro phosphorylated protein showed reduced condensate formation, thus indicating that the introduction of negative charges by mutation had effects similar to enzymatic phosphorylation^46^.

Due to the RNA binding ability of GRP7, Cy3-labeled miRNA164 was used to test whether or not AtGRP7 can recruit RNA into the liquid droplets. In the presence of 0.5 µM miRNA, droplet formation of AtGRP7 (10 µM) still occurred and the RNA co-localized with the protein (Fig. 6). This behavior was observed for wildtype AtGRP7, AtGRP7^RGG^, AtGRP7^StoE^, AtGRP^YtoG^ and AtGRP^StoG^ but not for AtGRP7^short^ and AtGRP7^YtoE^ (Fig. 6). In addition to a small RNA, a long RNA (*AtGRP7 CDS* RNA) was tested and similar phase separation and co-localization behavior was observed (Supplementary Fig. S14). Phase separation assays with the RGG domain alone confirmed that it is essential for condensate formation and is sufficient to recruit small and long RNAs into the droplets (Fig. 6, Supplementary Fig. S14).

## Discussion

In this study, microscale thermophoresis (MST) was used to investigate the RNA binding of GRP7 in detail. RNA binding plays a major role in the biological functions of GRP7, like its involvement in alternative splicing and cold stress response^16,30^. Therefore, the binding affinities of GRP7 for different RNAs and RNA modifications were investigated. By labeling the RNA with Cy5-UTP, unlabeled proteins could be used to determine the binding affinities. Thereby, protein labeling or tagging with fluorescence dyes was circumvented to avoid that the fluorescence dye is interfering with protein folding or function. RNAs for MST measurements were selected based on different criteria. *AtGRP7*, *AtGRP8* and *AtCOR15A* were selected since they were found to be bound by GRP7 via iClip and RIP-seq experiments^31^. Other RNAs used were selected from RNAs found in sequencing data of BnGRP7 bound RNAs, for example *CP12-1* and *novel miR2* with high read counts and *THIOREDOXINH10* and *miR164* with lower read counts (Supplementary Tab. S1-S3). *BnPARCL*, *tRNA-met* and *GFP* RNAs were selected as not expected GRP7 targets. Additionally, the structures of RNAs were predicted and the influence of methylation was analyzed. To elucidate the role of the RGG-domain, two deletion mutants, GRP7^short^ (lacking the RGG-domain) and AtGRP7^RGG^ (consisting only of the RGG-domain) were generated. Furthermore, the proteins with mutations within the RGG-domain targeting all tyrosines (AtGRP7^YtoE^ and AtGRP7^YtoG^) or S132 and S139 (AtGRP7^StoE^ and AtGRP7^StoG^), were produced. It was expected, that the mutations to glutamic acid simulate a phosphorylation and thereby modulating the RNA binding and phase separation of GRP7, while the mutations towards glycine were tested as controls. In addition to the measurements of RNA-binding behavior, phase separation assays with all proteins with and without RNA were performed.

For a first overview of the RNA-binding capability of GRP, RNAs known to interact with AtGRP7, like *AtGRP7*, *AtGRP8* and *AtCOR15A*, were tested. All of them showed a high binding affinity of 62–182 nM (Fig. 1, Supplementary Tab. S5). Even *BnPARCL*, which has not been found as a natural binding partner, was bound with a high binding affinity (99.7 nM) (Fig. 1). With 9.17 µM ± 3.49 µM, GRP7 showed a lower binding affinity towards GFP. To allow a comparison to results from previous studies using EMSA, the binding affinity of GRP7 to a sequence of the 3′UTR of GRP7 also called 7-UTR-WT^19^ was measured. The K_d_ of 0.16 µM measured using MST was in good agreement with the K_d_ of 0.1 µM determined by EMSA^19^.

Another possible biological function connected to GRP7’s RNA binding ability is the long-distance transport of RNAs through the phloem, since GRP7 was found in phloem sap of different plant species^14,47^. Therefore, GRP7s ability to bind phloem RNAs was tested to asses if its RNA binding capability might have an impact on the selection of certain RNAs for long distance transport in phloem. Considering the amount of phloem sap needed, *B. napus* was used to collect high amounts of phloem sap instead of *A. thaliana*. Therefore, AtGRP7 homologue BnGRP7 was coupled to sepharose beads and incubated with total phloem sap RNAs of *B. napus*. As a result, BnGRP7 did not enrich small RNAs and only a few longer mRNAs were significantly enriched compared to the input total phloem RNA, but with log2fold changes below 2 (Supplementary Tab. S1–S3). Altogether, more than 40,000 mRNAs were found in the eluted RNA fractions. These results suggest that GRP7 has no or only a low selectivity for certain RNAs.

To further investigate GRP7 RNA binding selectivity for small as well as long RNAs, MST was used to unravel if the binding affinity is influenced by RNA length. Here, long RNAs showed a tendency to be bound with higher dissociation constants, thus lower binding affinities, while all small RNAs tested showed lower dissociation constants in the nanomolar range (Fig. 2). Such a difference in binding affinity between long and short RNAs was only observed in the present study, since previous publications often concentrated on measuring short oligonucleotides only^11,16,19,20^. This difference in binding affinities for small and long RNAs was significant for two of the long RNAs (*defensin-like-protein 3* and *Notchless protein*) (Fig. 2). In comparison to other long RNAs with higher binding affinities, like *AtCOR15A pre-mRNA* and *AtGRP7 pre-mRNA*, the two RNAs with lower binding affinities showed more structure according to in silico RNA folding predictions by RNAfold 2.6.3^48,49^ (Supplemental Fig. S7). Furthermore, in silico structure analysis showed that *GFP*-RNA is more structured as well. Therefore, the structure might be one factor causing the lower binding affinity of GRP7 towards these RNAs. On the other hand, GRP7 had a similar binding affinity for *AtGRP7* CDS that showed a moderate structure as for the less-structured *AtGRP7 pre-mRNA*. To further analyze if RNA structure influences GRP7 binding affinity, the G-quadruplex RNA *Sc1* was used. As LiCl resolves secondary structures of RNA like G-quadruplexes^50,51^, MST was performed with standard MST buffer and buffer containing LiCl instead of NaCl. GRP7 showed a significantly higher binding affinity to *Sc1*-RNA with LiCl (K_d_ 0.463 µM ± 0.001 µM) compared to *Sc1*-RNA in standard MST-buffer (K_d_ 6.9 µM ± 4.39 µM) (Supplementary Fig. S8), suggesting that GRP7 preferentially binds less structured RNAs. Nevertheless, GRP7 bound all RNAs tested. A similar behavior was observed for the FUS-protein, which participates in alternative splicing of RNA, similar to GRP7, and is a partially disordered protein with RGG-domains and a structurally conserved RRM^52,53^. The FUS protein bound a wide range of different RNAs and did not show a huge difference in binding affinity not even compared to a negative control^54^. FUS did not show preference to a specific sequence motif but it is proposed to recognize RNA with a mixture of shape and sequence specificity^55^. Other similar proteins like hnRNP showed a broad RNA binding spectrum as well^56–58^. On the other hand, GRP7 selectivity for certain mRNAs was suggested by iCLIP and RIP-seq experiments, where 452 bound mRNAs were found^31^. GRP7 bound to the mRNAs preferably in the UTR-regions but also in exons and introns, and a favored UC-rich sequence motif was identified^31^. In the more than 40,000 phloem RNAs bound by GRP7 in this study (Supplementary Tab. S1), a similar UC-rich motif could not be confirmed by a motif search. Therefore, our results suggest that GRP7 alone is, like FUS and hnRNP, a protein without a clear binding selectivity. However, selectivity for certain RNAs in vivo could be modulated by other factors like cellular localization, RNA abundance, or interaction partners.

Comparing single stranded and double stranded RNAs, GRP7 showed a preference for single stranded RNAs (Fig. 2c) which is in line with previous findings of the interaction of RRM domain with ssRNA^2^.

By now, GRP7 was not reported to bind methylated RNAs. In the present study, GRP7 was shown to bind methylated RNA, but without a significant preference for m6A or m5C methylated RNAs. These RNA modifications seem to have no major influence on GRP7 RNA binding (Fig. 3).

Previously, a truncated version of GRP7 only containing the RRM showed weaker interactions with RNA compared to the full-length protein^11^. Hence, the RGG-domain could be potentially involved in RNA binding. Considering the dispersed amino acids arginine, tyrosine and serine within the RGG-domain, this disordered region has similarities to RGG-domains and other intrinsically disordered regions that showed RNA binding capability^59–61^. As reported, truncated GRP7 (GRP7^short^) only showed weak binding for small RNAs and no binding for longer RNAs (Fig. 4). Since previous studies used short oligonucleotides only^11^, this difference was not observed before for GRP7. However, a lower RNA binding affinity was also observed for another protein, nucleolin, lacking the RGG-domain. Here, the truncation led to a tenfold decrease of its RNA binding affinity compared to wild type nucleolin. Both, the RRM- and RGG-domains were required for efficient complex formation with pre-rRNA^62^. Moreover, the RGG-domain of the FUS protein significantly increased the affinity of the RRM for certain nucleobase sequences and especially nucleotide structures^55,60^. Thus, the RGG-domain of GRP7 might have a similar effect, changing the secondary structures especially of longer RNAs and thereby providing the possibility for GRP7 to bind to RNAs with higher affinity and to form RNA–protein complexes with higher stability. To examine if the RGG-domain is capable of binding RNA on its own, AtGRP7^RGG^ was produced. Due to its high tendency to form condensates and aggregates, this protein could only be purified with the N-terminal eYFPtag. As a consequence, eYFP-AtGRP7^RGG^ was used as the labeled interaction partner with a stable concentration and RNA was titrated. As a control, eYFP alone was measured to exclude that the tag binds RNA (Supplementary Fig. S9). eYFP-AtGRP7^RGG^ was able to bind the small *GRP7 3’UTR* RNA as well as the long *AtGRP7 CDS* RNA (Fig. 4d), while AtGRP7^short^ was only able to bind small RNAs but no long RNAs (Fig. 4b, c). This underlines the importance of AtGRP7s RGG-domain for RNA binding, especially of long RNAs.

Mutations of tyrosine to glutamic acid (GRP7^YtoE^), simulating possible phosphorylation within the RGG-domain by the introduction of a negative charge, resulted in significant weaker binding affinities for longer RNAs (Fig. 5), while small RNAs were bound with similar binding affinities compared to the WT protein (Supplementary Fig. S11). GRP7^YtoG^ showed lower binding affinities for longer RNAs, but not as prominent as the GRP7^YtoE^ mutant (Fig. 5). These results indicate that the interaction of the RGG-domain seems to be more important for long RNA binding of GRP7 than for binding small RNAs and that the different types of amino acids used for the exchange had different effects. For a modified RGG-domain of TLS/FUS it was shown that the tyrosines within the RGG-domain facilitate the recognition of 2′-OH of ribose in G-quadruplex structures^63^. Hence, it could be possible that the mutation of Y to E in GRP7 disrupts interactions of the RGG-domain with ribose in longer RNAs, thereby reducing the binding affinity of GRP7^YtoE^ towards longer RNAs in addition to probable repulsion of negative charged RNA by the negatively charged glutamic acid. Additionally, for an A/B type hnRNP it was shown that phosphorylation of tyrosine within a similar glycine-rich region reduced the binding affinity towards poly-U RNA and abolished binding of poly-A RNA^64^. A similar effect might be true for the phosphomimic variant GRP7^YtoE^. Mutations of two serines (S132 and S139) within the glycine-rich region either towards glutamic acid or glycine had no major influence on GRP7 RNA binding ability. Preceding work identified the phosphorylation of S132 and S139 *in planta* and showed that inhibition of GRP7 phosphorylation reduced nuclear localization of GRP7^38^. Considering the involvement of GRP7 in alternative splicing and transport of RNA from the nucleus into the cytoplasm, phosphorylation leading to nuclear localization should not weaken the interaction of GRP7 with RNA to further provide its full function. For this reason, similar binding affinities of AtGRP7^StoE^ and AtGRP7 could support this hypothesis. Previous studies on the FUS protein showed only minimal changes in binding affinity of RNAs bound with high affinity when mutating its RGG-domain, while larger changes in dissociation constant only occurred for RNAs that were bound with lower affinities before^60^.

Considering that some differences in binding affinity between AtGRP7 and BnGRP7 were observed (Figs. 2, 5, Supplementary Fig. S4), the RGG-domain was considered a possible reason for this difference. Hence, AtBnGRP7 was generated. The analysis of AtBnGRP7 RNA binding did not show any significant differences in dissociation constants between AtGRP7 and AtBnGRP7 for RNAs tested (Fig. 5). Thus, the results suggest that the RGG-domain alone is not determining the binding affinity, but the whole protein including RRM and RGG-domain.

Phosphorylation of tyrosines within IDRs was reported to influence phase separation behavior^65^. Considering the similarity of the RGG-domain from GRP7 with other IDRs and that GRP7 can form condensates^44^, the phase separation behavior of WT GRP7 and all mutants was investigated. AtGRP7^WT^ as well as AtGRP7^short^, AtGRP7^RGG^, AtGRP7^YtoE^, AtGRP7^StoE^, AtGRP7^YtoG^ and AtGRP7^StoG^ were tagged with eYFP at the N-terminus. Phase separation was visible for AtGRP7^WT^ with and without RNA (Fig. 6, Supplementary Fig. S14). The RNA was recruited into the liquid droplets formed by AtGRP7 (Fig. 6, Supplementary Fig. S14). A lower number of liquid droplets was observed for AtGRP7^StoE^ without RNA and even less with RNA. For AtGRP7^YtoE^ and AtGRP7^short^, almost no droplets formed, while AtGRP7^RGG^, AtGRP7^YtoG^ and AtGRP7^StoG^ showed liquid droplets (Fig. 6). Therefore, the introduction of negative charge at the tyrosines, like a phosphorylation would, reduced the ability of GRP7 to form condensates and not the exchange of tyrosine itself. It is likely, that the phase separation of GRP7 is regulated by phosphorylation since it was already shown that GRP7 can be phosphorylated by RALF-FERONIA in vitro and *in planta*^38^. Besides AtGRP7, AtPARCL can be phosphorylated by RALF-FERONIA. For AtPARCL, a reduced liquid condensate formation was observed, when negative charges were introduced in the IDR in form of Y to E exchanges or phosphorylation of tyrosines as it was for AtGRP7^46^. By this mechanism, the condensation formation of AtPARCL was modulated while the RNA binding was not influenced by this modification^46^. This is in contrast to AtGRP7, for which the Y to E exchange not only modulated its phase separation behavior but also reduced its RNA binding capacity (Figs. 5 and 6).

Taken together, our results demonstrate that GRP7 has a broad capacity to bind RNA molecules with diverse sequences, structures, lengths, and modifications. The RGG-domain of GRP7 plays a crucial role in facilitating phase separation, and the mutation of the amino acids Y to E within the RGG-domain negatively affects phase separation. Besides this, the RGG-domain is important for AtGRP7's RNA binding capability, particularly towards longer RNAs. Moreover, the RGG-domain is sufficient to bind RNA and to recruit RNA to condensates even without the RNA-binding domain. This underlines the importance of not only investigating the typical RNA-binding domains of a protein, but to also take other domains into account, as they can have a huge impact on RNA-binding.

## Material and methods

### Growth of plants

*Brassica napus* cv. Drakkar plants were grown on soil in the greenhouse at 16 °C with a light/dark cycle of 16 h/8 h.

*Arabidopsis thaliana* Col-0 plants were grown on soil at 22 °C with a light/dark cycle of 16 h/ 8 h in a climate chamber.

### Phloem sap sampling

The phloem sap of *B. napus* was sampled during noon as previously described^66^. In brief, eight-week-old plants which showed an inflorescence but did not start to flower yet were watered before sampling and punctured at the inflorescence stem multiple times. The first drop of all punctuations was removed and all following drops were collected in a pre-cooled 1.5 ml reaction tube. The collection was carried out for around an hour and sampled phloem sap was frozen in liquid nitrogen and stored at − 80 °C.

### Genomic DNA isolation

Genomic DNA was isolated with Plant Genomic DNA Mini Kit (Avegene) according to manufactures instructions. Genomic DNA was eluted with 50 µl H_2_O.

### RNA isolation from phloem sap and leaf material and cDNA synthesis

RNA was isolated from leaf material of *B. napus* as well as *A. thaliana* and from phloem sap of *B. napus*. Leaf material was frozen in liquid nitrogen and ground. 100 mg of ground leaf material was used subsequently for RNA isolation.

RNA isolation was performed with TRIzol™ (Thermo Fisher Scientific) for leaf material or TRIzol™-LS (Thermo Fisher Scientific) for phloem sap in combination with RNA Clean & Concentrator-25 RNA-Kit (Zymo Research) according to manufacturer’s protocol. The aqueous phase from TRIzol preparations was then used as input for the RNA Clean & Concentrator-25 RNA-Kit (Zymo Research).

cDNA synthesis was done with 150–1000 ng of RNA per reverse transcriptase reaction with RevertAid from ThermoFisher according to the manufacturer’s protocol.

### Cloning of expression constructs

For cloning the expression constructs, pET28a+ (Novagen) or a modified GoldenGate compatible pET28a+ has been used. To clone AtGRP7, the gene was amplified from A. thaliana cDNA with primers including restriction enzyme recognition and cutting sites of NdeI (fw primer) and NotI (rev primer) and inserted into pET28a+ with the 6xHis-tag and thrombin recognition site at the N-terminus in frame with the gene. BnGRP7 was cloned in frame with N-terminal 6xHis-tag and TEV-recognition site into the modified GoldenGate compatible pET28a+ using BsaI. For AtGRP7^short^, a side directed mutagenesis was conducted on the pET28a+ AtGRP7 construct to mutate amino acid 88 to a stop codon. For AtBnGRP7 mutant, the RRM of AtGRP7 and the glycine-rich domain of BnGRP7 were amplified with primers including BsaI cutting and recognition sites for seamlessly cloning into pET28a+ modified GoldenGate compatible vector in frame with N-terminal 6xHis-tag and TEV-recognition site. AtGRP7^RGG^ was cloned into pet28a+ containing eYFP by amplifying the RGG-domain with primers containing the restriction enzyme sites for BamHI and XhoI as well as the recognition site for thrombin, to allow the removal of eYFP after expression.

For AtGRP7^YtoG^, AtGRP7^YtoE^, AtGRP7^StoG^ and AtGRP7^StoE^, the glycine-rich domain with mutations was synthesized by Eurofins Genomics. The wildtype RRM of AtGRP7 was amplified with primers including BsaI recognition sites and cloned seamlessly in frame with the synthesized glycine-rich domains using BsaI. The constructs were cloned in frame into pET28a+ modified Golden Gate compatible vector with N-terminal 6xHis-tag and thrombin recognition site.

For constructs with AtGRP7, AtGRP7^short^, AtGRP7^YtoE^, AtGRP7^YtoG^, AtGRP7^StoE^ and AtGRP7^StoG^ with eYFP, a modified pET28+ containing an eYFP was used. The genes were cloned with the eYFP at the N-terminus in frame by adding BamHI and XhoI restriction enzyme sites by PCR to the amplicons of AtGRP7 and the mutants.

### Expression and purification of AtGRP7, AtGRP7 mutants and BnGRP7

AtGRP7, AtGRP7 mutants and BnGRP7 were expressed in BL21 (DE3) RIPL *E. coli* expression strain. Two 400 ml expression cultures using ZY-autoinduction media^67^ were grown at 37 °C for 2–3 h, then shifted to 24 °C and grown over night. The expression culture was centrifuged and the pellets either frozen for later use at − 20 °C or directly used for lysis.

Lysis was conducted for 20 min at RT using 50 ml lysis buffer containing 50 mM HEPES pH 8.0, 200 mM KCl, 3% glycerol, 20 mM Imidazole, 100 µg/ml Lysozyme, 1 tablet of Protease inhibitor (Roche), 1 mM PMSF, 1 mM AEBSF and 1 mM DTT. Following, sonification was conducted on ice with a sonicator (Branson Sonifier 250). The lysis was centrifuged at 40,000 × g and the supernatant was filtered through a 0.45 µM syringe filter. The supernatant was then loaded onto a HisTrap™ Fast Flow column (Cytiva), washed with 5 column volumes lyse buffer with high salt concentration (50 mM HEPES pH 8.0, 1 M KCl, 20 mM Imidazole, 3% glycerol, 1 mM DTT) followed by 5 column volumes of lyse buffer (50 mM HEPES pH 8.0, 200 mM KCl, 3% glycerol, 20 mM Imidazole, 1 mM DTT). Bound protein was eluted on the ÄKTA^TM^start (GE-Healthcare) with an imidazole gradient of 15 column volumes from 0.02 M up to 1 M imidazole. Fractions were selected which had a high UV-absorption at 260 nm and analyzed on an SDS-gel. Fractions with high amounts of the protein of interest were pooled and dialyzed with 20 U thrombin for 2 h at RT against 1 l dialysis buffer (25 mM HEPES pH 7.0, 300 mM KCl, 3% glycerol, 1 mM DTT). A reverse nickel column was performed to remove residual His-tagged protein and the flow through was concentrated down to 5 ml following a size exclusion chromatography with HiLoad Superdex 16/600 75 pg. Protein containing fractions were analyzed on an SDS gel and fractions containing the protein were pooled and concentrated in a Vivaspin50 concentrator MWC 5,000 or MWC 10,000 up to 1.6–2.0 mg/ml protein.

### BnGRP7 coupling to CnBr sepharose beads

To couple BnGRP7 on CnBr-activated Sepharose™ 4B beads (GE Healthcare), 500 µg of protein and 40–50 mg of CnBr-sepharose™ 4B beads were used. The coupling was done according to manufacturer’s instructions. In short, Sepharose™ beads were washed three times with 300 µl of 1 mM HCl. Following the first and second washing step, beads were centrifuged for 30 s at 3000 × g and the supernatant was removed. After the third washing step, the beads were transferred to a microcentrifugation spin column and removal of supernatant was done by centrifugation for 1 min at 700 × g. 500 µg BnGRP7 and 1 mg dextran sulfate with 250 µl coupling buffer (0.1 M NaHCO_3_ pH 8.3, 0.5 M NaCl) were added to the beads and incubated over night at 4 °C rolling. Supernatant was removed by centrifugation for 1 min at 700 × g and 4 °C. By washing the beads twice with coupling buffer, any remaining unbound protein was removed. 400 µl blocking buffer (0.1 M Tris–HCl, pH 8.0) was used to block any remaining uncoupled sites by rolling incubation over night at 4 °C. Subsequent, supernatant was removed by centrifugation for 1 min at 700 × g and 4 °C.

As negative control, the beads were treated the same way as described above without adding protein during coupling.

### RNA binding of immobilized BnGRP7

To assess the RNA-binding capability of immobilized BnGRP7, 25 µg total phloem RNA was utilized. After storing at -80 °C, the beads underwent thawing on ice and three washes with assay buffer (25 mM HEPES pH 7.5, 150 mM sodium acetate) using a microcentrifuge spin column. Subsequently, 200 µl assay buffer and 25 µg of RNA were added to the beads, followed by an incubation of 10 min at room temperature with rolling. The flow through was collected via centrifugation at 4 °C and 700 × g for 30 s in a clean 1.5 ml reaction tube. The beads underwent five washes with 300 µl assay buffer, and each washing step was collected in 1.5 ml tubes through centrifugation at 4 °C and 700 × g for 30 s.

Elution for RNA was performed in three steps, beads were first incubated with assay buffer containing 250 mM sodium acetate for 5 min at room temperature, and the elution was collected as described earlier. The second elution mirrored the first, with assay buffer containing 500 mM sodium acetate. After collecting the second elution, the third elution was conducted similarly with assay buffer containing 2 M sodium acetate and was collected through centrifugation.

To increase the salt concentration to 300 mM for precipitation, assay buffer with 2 M sodium acetate was added to the flow through, washing steps, and the first elution. For RNA precipitation, 2.5–3 volumes of 100% ethanol were added to the flow through, washing steps, and elution fractions. After incubation over night at − 20 °C, precipitated RNA was centrifuged for 30 min at 20,000 × g and 4 °C. The pellet was washed with 70% EtOH and centrifuged for 5 min at 7500 × g. The supernatant was removed and the pellet was air-dried and resuspended in 50 µl H_2_O.

### RNA sequencing

A quality check of RNA send for sequencing was performed through bioanalyzer as well as nanodrop. Phloem RNA was additionally checked for its purity by performing a PCR targeting the small RuBisCO subunit and ThioredoxinH. RNA that was qualified for RNA sequencing was send to Novogene (Cambridge, UK) for subsequent RNA sequencing library preparation and Illumina sequencing as well as the bioinformatic analysis.

### In vitro transcription of short and long RNA

For in vitro transcription of long RNA, template DNA was prepared by amplifying the target gene with a forward primer containing T7 promotor sequence and two additional guanines, if the target sequence did not start with two guanines. As template, DNA, cDNA or a plasmid, if the target was cloned into pet28a+ or pUC57, were used. The PCR was purified with NucleoSpin® Gel and PCR Clean-up kit (Macherey–Nagel) or Monarch® DNA Gel extraction kit (New England Biolabs). The template DNA for small RNA (< 100 nt) was prepared by annealing of two complementary primers containing T7 promotor sequence at 95 °C for 5 min and cooldown at RT.

For RNA synthesis, an already published protocol was used and adapted^68^. In short, 5 pmol of DNA template, 5 µl of inorganic pyrophosphatase (0.1 U/µl), 25 U/µl T7 polymerase (in house purified), 10% DMSO (final concentration, only for longer RNA), 10 µl 10× reaction buffer (50 mM Tris–HCl pH 7.5, 15 mM MgCl_2_, 5 mM DTT and 2 mM spermidine), 1 U/µl RiboLock (Thermo Fisher Scientific) and 2 mM of each NTP were used. To add Cy5-or Cy3-labeled UTP, 0.05 mM to 0.25 mM Cy5- or Cy3-labeled UTP was added to the reaction and the concentration of non-labeled UTP was adjusted accordingly. To obtain methylated RNAs, 1 mM m5CTP and m6ATP was combined with 1 mM non-methylated NTP. The in vitro transcription was conducted for 2 h up to overnight at 37 °C or 30 °C, depending on the length of RNA transcribed (longer transcripts were usually incubated at 30 °C). The reaction was stopped with 5 mM EDTA and RNA was purified using the RNA Clean & Concentrator-25 RNA-Kit (Zymo Research) according to manufacturer’s protocol. For *AtGRP7*, *AtGRP8* and *AtCOR15A* transcripts with and without UTR, T7-RNA Polymerase from Thermo Fisher Scientific was used according to manufacturer’s protocol, incubated for 2 h at 37 °C and RNA was purified as described before.

### Microscale thermophoresis measurements

For MST-measurements, RNA was labeled during in vitro transcription with Cy5 labeled UTP. Titration series were prepared with the unlabeled protein using MST buffer (25 mM Tris–HCl pH 8.0, 150 mM NaCl, 1 mM DTT and 0.1 mg/ml BSA) and a starting concentration of 50–75 µM. The fluorescently labeled compound, the RNA, was added 1:1 with a final concentration of 20 nM for RNA. Everything was mixed well by pipetting and centrifuged down in a microcentrifuge. Protein and RNA were incubated for around two minutes. Monolith™ Series standard capillaries (Nanotemper technologies, Germany) were used to load 16 samples and placed in the sample slide of the Monolith NT.115 MicroScale Thermophoresis device (Nanotemper technologies). Binding affinity measurements were performed using the autofluorescence detection setting and medium MST-power for the first measurement of each individual RNA and using the fluorescence setting selected by the autodetection from the first measurement for the following measurements. Each protein was measured with each RNA at least in three repetitions. For each repetition a titration series was prepared.

For eYFP-AtGRP7^RGG^, the titration series were prepared with unlabeled RNA with starting concentrations of 7.5–9.5 µM and a constant final concentration of protein of 20 nM. The measurements were performed as described for unlabeled protein and labeled RNA.

All repetitions were analyzed separately for their dissociation constant (K_d_) with MO.Affinity Analysis software (v2.1.2) and measurements with signal to noise ratio of ≥ 5 as well as response amplitude ≥ 5 were accepted as binding event. Everything below this threshold was regarded as no binding. Means and standard deviation were calculated with Origin 2021b (OriginLab) v9.8.5.201. Normal distribution of data was tested with Shapiro–Wilk test for p = 0.01 with Origin 2021b (OriginLab) v9.8.5.201. Comparisons between binding affinities were conducted with one-way ANOVA and Tuckey test with Origin 2021b (OriginLab) v9.8.5.201 for p = 0.05, as indicated in the descriptions of bar graphs.

### In vitro test for liquid–liquid phase separation

The eYFP tagged protein was mixed with phase separation buffer (50 mM Tris–HCl pH 7.5 at RT, 150 mM NaCl) with and without 0.5 µM RNA and a protein concentration of 10 µM. After 10 min of incubation at RT, 10% PEG3350 was added and followed by 15 min incubation on ice. Phase separation was investigated with Keyence BZ-X800E microscope and YFP and RFP filters.

### Plants guidelines

The authors confirm that experimental research and field studies on plants (either cultivated or wild), including the collection of plant material, comply with relevant institutional, national, and international guidelines and legislation.

### Material statement

All plant material was obtained at Universität Hamburg, Germany and no specific permissions or licenses are required as it is our own material.

### Supplementary Information

Supplementary Information 1.

Supplementary Tables.

## Data Availability

All Materials described in the manuscript will be freely available to any researcher wishing to use them for non-commercial purposes. Request should be directed to the corresponding author. Raw Illumina Sequencing Data will be available at UHH-FDR 10.25592/uhhfdm.13976.
