# Supplementary material for: The glycine-rich domain of GRP7 plays a crucial role in binding long RNAs and facilitating phase separation
Source: Sci Rep. 2024 Jul 11;14:16018. doi: 10.1038/s41598-024-66955-5 (PMC11239674; doi:10.1038/s41598-024-66955-5)
Supplement: Supplementary file 1 — Supplementary Information 1. [file 41598_2024_66955_MOESM1_ESM.docx]

**Supplementary Material**


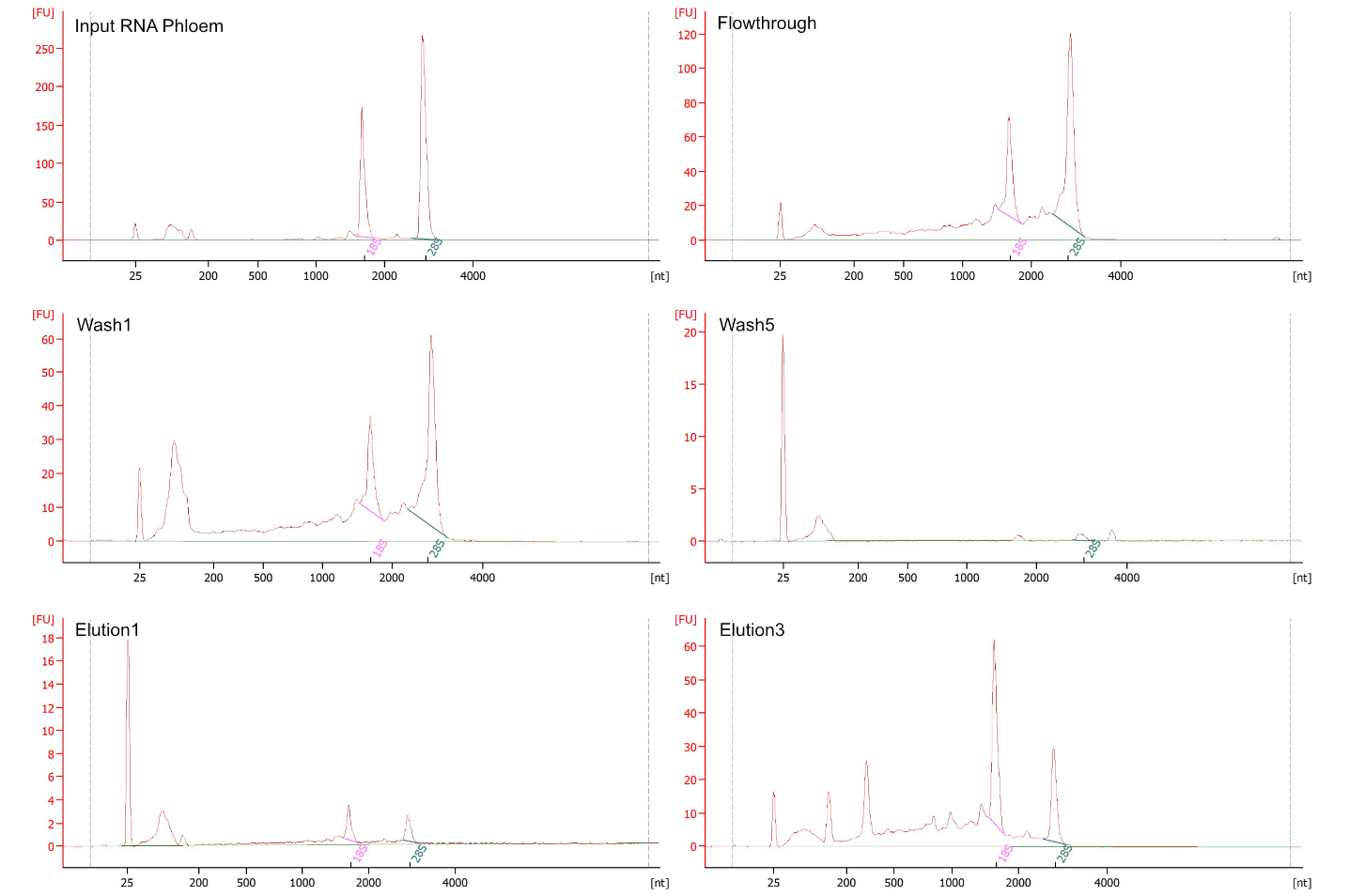


**Supplementary Figure S1: Bioanalyzer electropherograms total phloem RNA used as input and RNA bound by BnGRP7 coupled to CnBr Sepharose.** The electropherograms show the fluorescence units (FU) on the Y-axis, representing a measure of how much RNA was detected while the X-axis represents the length of RNA in nucleotides (nt).


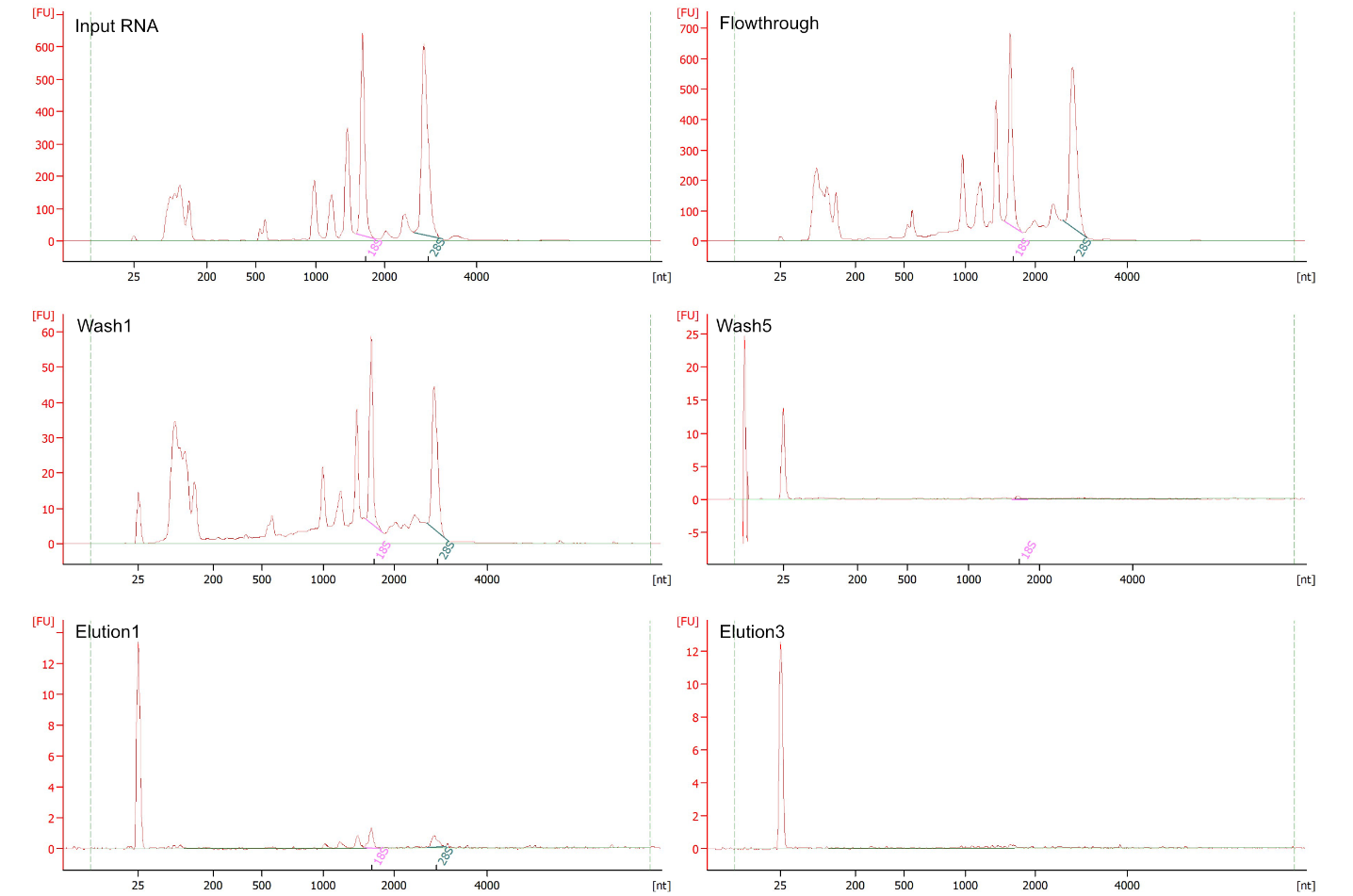


**Supplementary Figure S2: Bioanalyzer electropherograms total phloem RNA used as input and RNA bound by negative control (no protein coupled to CnBr Sepharose).** The electropherograms show the fluorescence units (FU) on the y-axis, representing a measure of how much RNA was detected while the x-axis represents the length of RNA in nucleotides (nt).


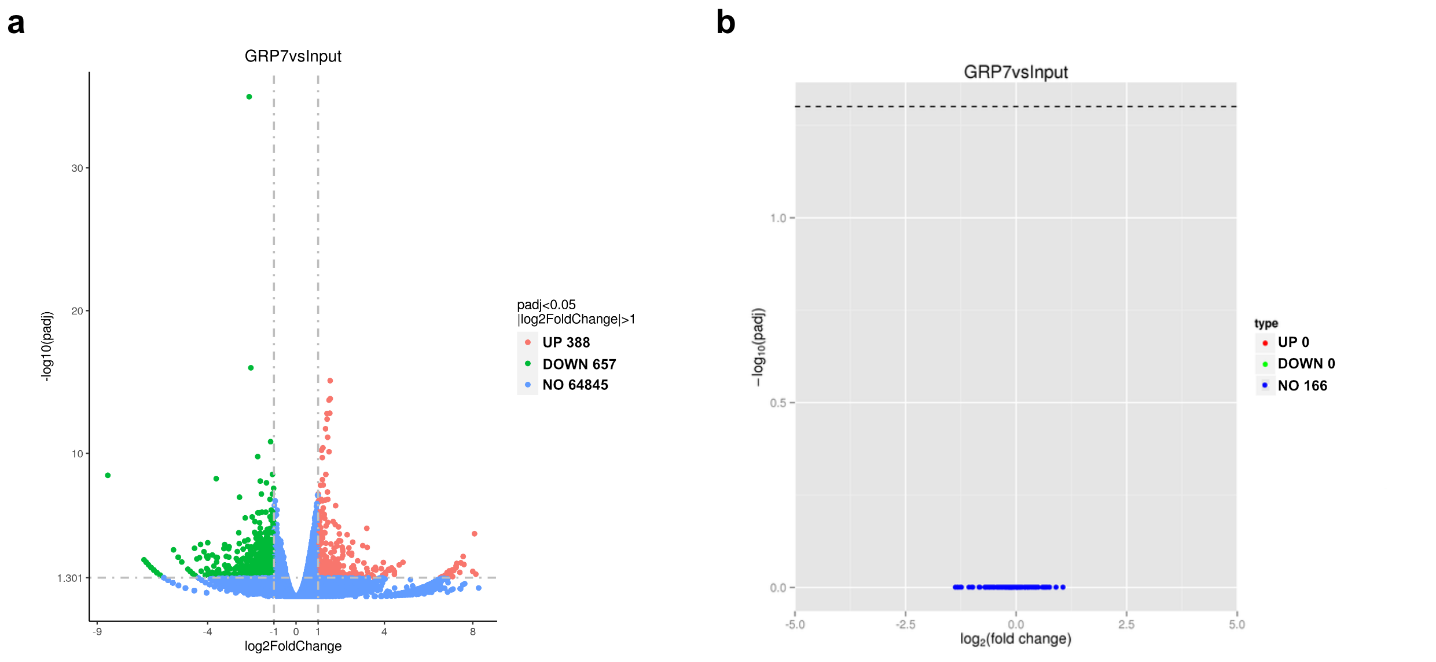


**Supplementary Figure S3: Volcano plots phloem RNA sequencing.** a) Volcano plot mRNA sequencing, comparison of eluted RNAs from coupled BnGRP7 and input phloem RNA. b) Volcano plot small RNA sequencing, comparison of eluted small RNAs from coupled BnGRP7 and input phloem RNA.


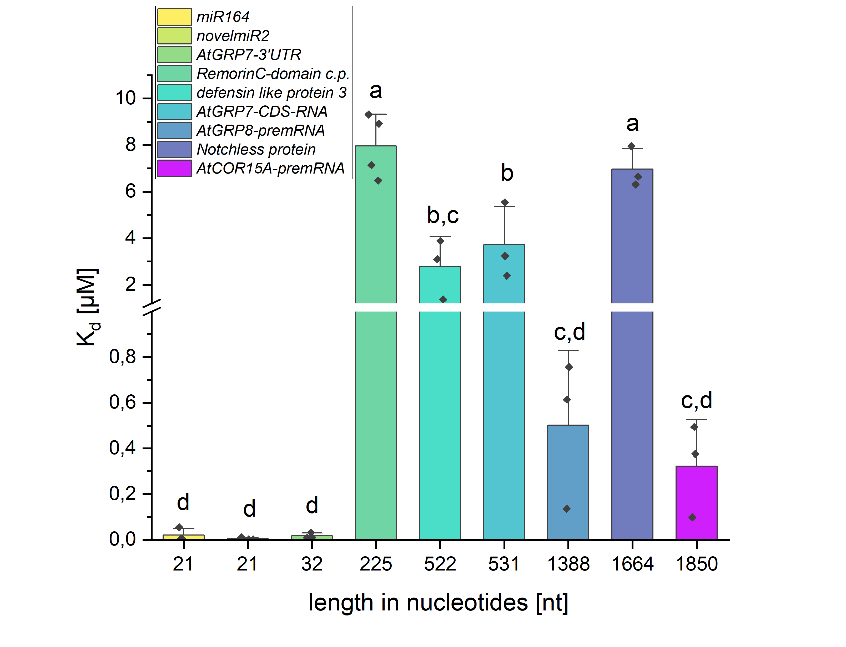


**Supplementary Figure S4: Dissociation constants (K_d_s) of BnGRP7 towards RNAs with different length.** The binding affinity of BnGRP7 towards the different RNA was compared by a one-way ANOVA with a Tukeys test (p= 0.05). RNAs which don’t share the same letter have significant different K_d_s. Y-axis: K_d_ in µM, cut between 1 and 1.2 µM, X-axis: RNA-length in nt.


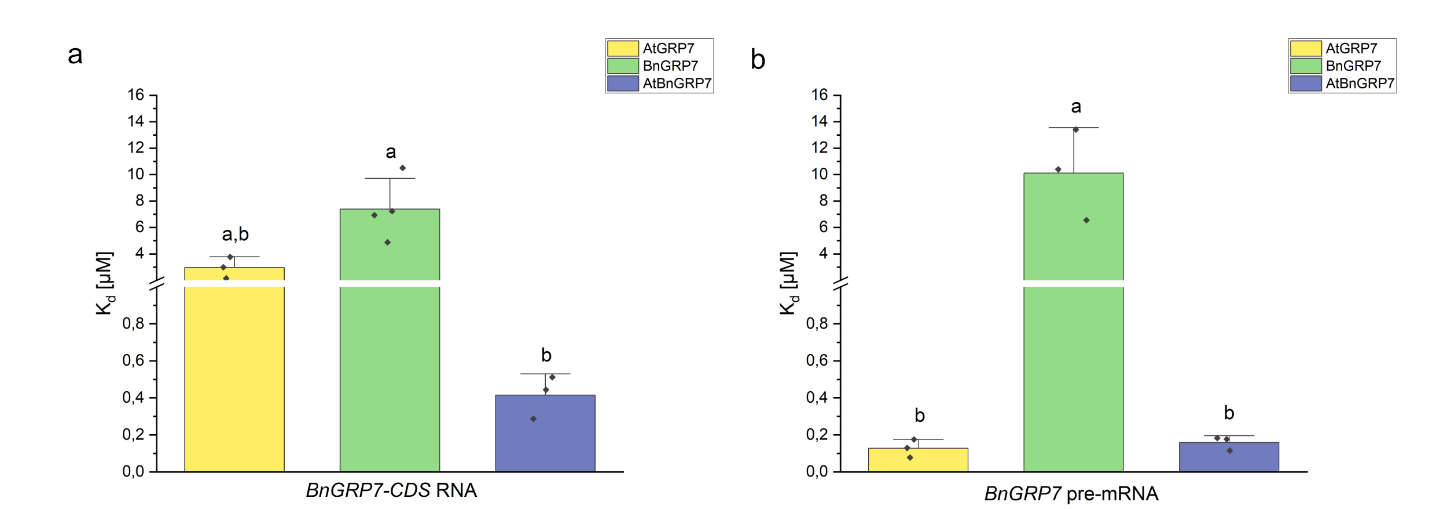


**Supplementary Figure S5: Dissociation constants (K_d_s) of AtGRP7, BnGRP7 and AtBnGRP7 for BnGRP7-CDS RNA and BnGRP7-premRNA. a)** The binding affinity of AtGRP7, BnGRP7 and AtBnGRP7 towards BnGRP7-CDS RNA was compared by a one-way ANOVA with a Tukeys test (p=0.01). RNAs which don’t share the same letter have significant different K_d_s. Y-axis: K_d_ in µM. **b)** The binding affinity of AtGRP7, BnGRP7 and AtBnGRP7 towards BnGRP7-premRNA was compared by a one-way ANOVA with a Tukeys test (p=0.01). RNAs which don’t share the same letter have significant different K_d_s. Y-axis: K_d_ in µM, cut between 1 and 2 µM .


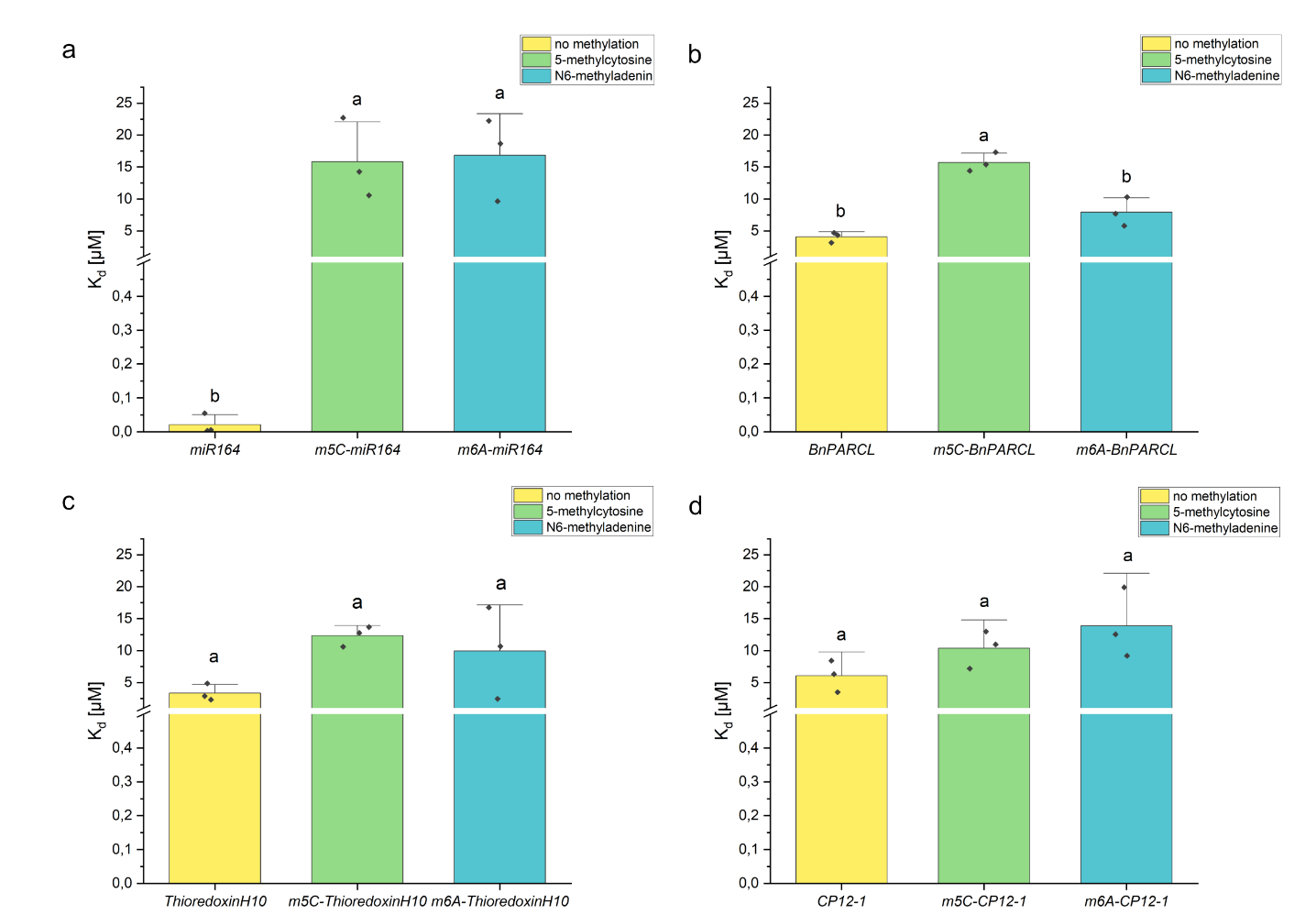


**Supplementary Figure S6: Comparison of the K_d_s of BnGRP7 for unmethylated and methylated RNAs. a-d)** display the binding affinity of BnGRP7 for four different RNA with and without methylations. Methylated RNA contained around 50 % 5-methylcytosine or 50 % N6-methyladenine. Y-axis: K_d_ in µM, cut between 0.5 and 1 µM , X-axis: different RNA with different methylations. The binding affinities of BnGRP7 for methylated and unmethylated RNA were compared with a one-way ANOVA and a Tukey-test for p=0.05. Shared letters indicate no significant difference in binding affinity.


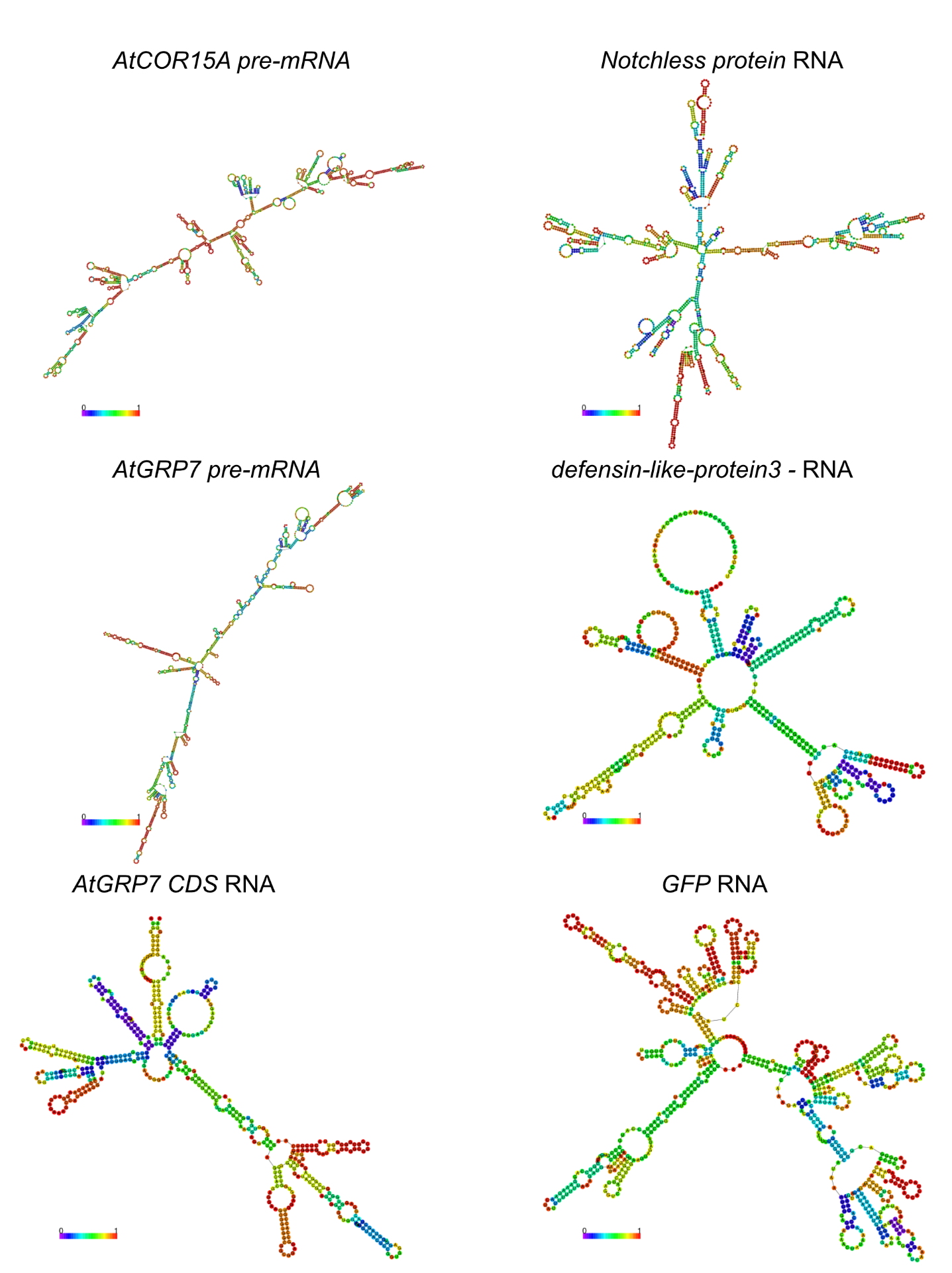


**Supplementary Figure S7: In-silico predicted minimal free energy (MFE)** **RNA structures of different RNAs used in MST.** The colors of the bases display the base-pairing probabilities, while it displays the probability of being unpaired for unpaired bases. For the prediction of the RNA structures, RNAfold^66-67^ was used.


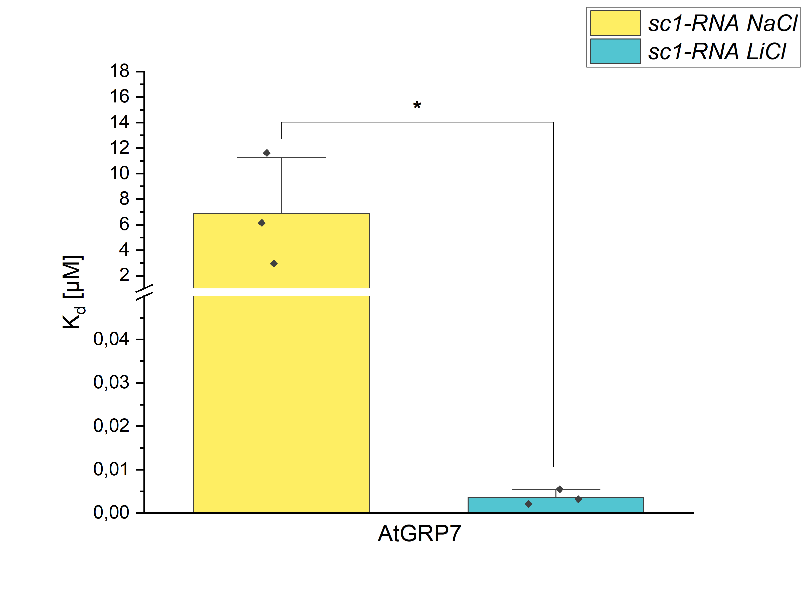


**Supplementary Figure S8: Dissociation constants (K_d_) of sc1-RNA in NaCl and LiCl buffer for AtGRP7.** The y-axis shows the K_d_ in µM. Sc1-RNA was measured in standard MST buffer (NaCl) and MST buffer containing LiCl instead of NaCl for its binding affinity for AtGRP7. With a students t-test and p=0.05, the binding affinity of sc1 NaCl is significantly different compared to the binding affinity of sc1 LiCl.


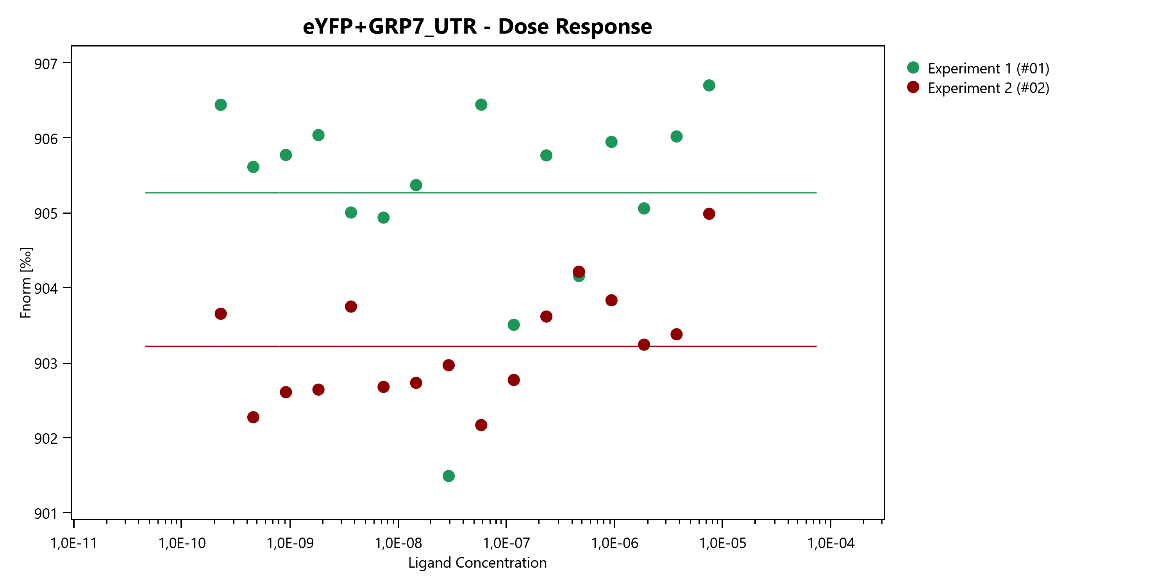


**Supplementary Figure S9: Dose response curve of eYFP with AtGRP7 3’UTR RNA**. Change in fluorescence plotted against the ligand concentration. The x-axis displays the ligand concentration (in this case AtGRP7 3’UTR RNA) and the y-axis the Fnorm (‰), the change in fluorescence. No binding of eYFP and the RNA was detected, as the Fnorm did not show a significant increase or decrease along the different ligand concentrations.


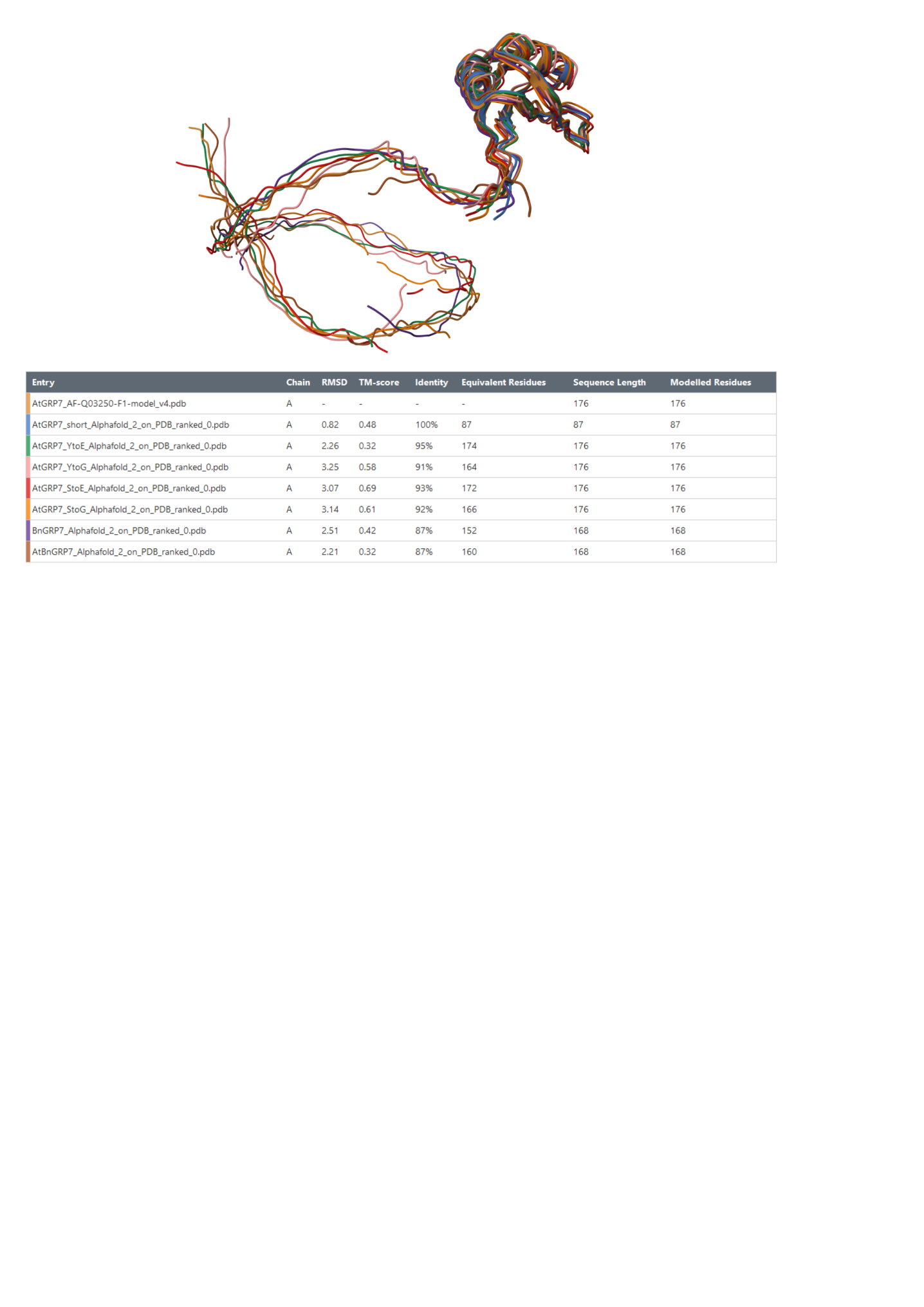


**Supplementary Figure S10: Structure alignment of AtGRP7, BnGRP7 and mutant version.** The predicted Alphafold structures of AtGRP7, BnGRP7 and different mutants were aligned with the pairwise structure alignment tool of RCSB PDB using the jFATCAT-flexible alignment algorithm.


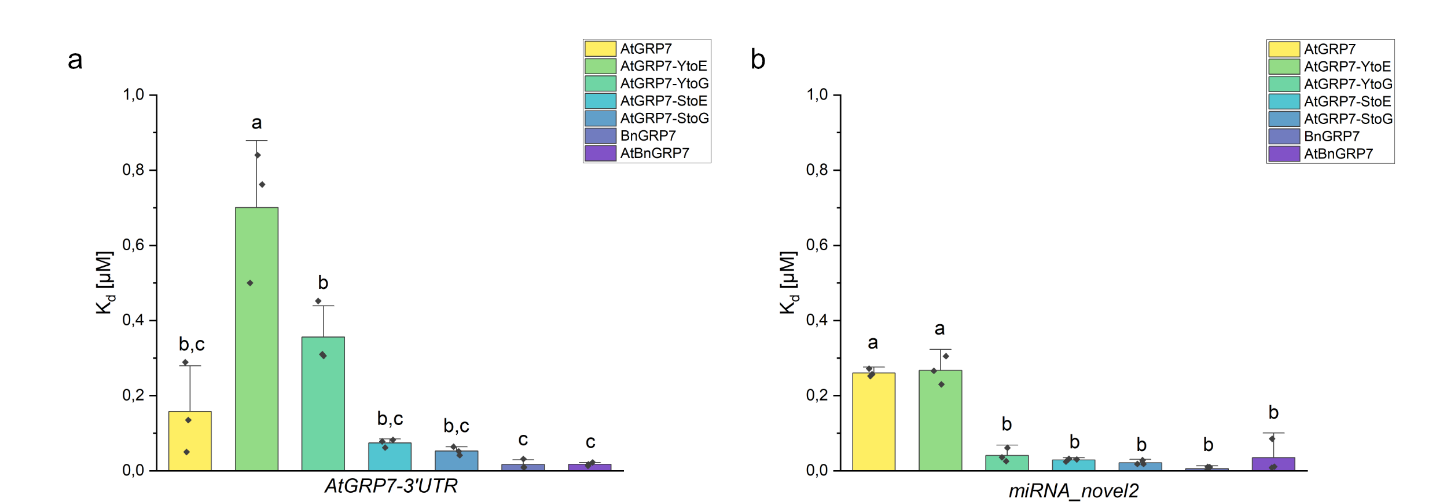


**Supplementary Figure S11: Dissociation constants (K_d_s) of AtGRP7, BnGRP7 and different mutants for small RNAs. a)** RNA binding affinities of AtGRP7, AtGRP7-YtoE, AtGRP7-YtoG, AtGRP7-StoE, AtGRP7-StoG, BnGRP7 and AtBnGRP7 for the 32 nt long binding site of AtGRP7-3’UTR. **b)** RNA binding affinities of AtGRP7, BnGRP7 and mutants for miRNA_novel2 (similar to bna-miR6030). Y-axis shows the K_d_ in µM while the X-axis displays the RNA measured. The boxes in different colors represent the K_d_ of the different proteins for the RNA displayed on the x-axis.


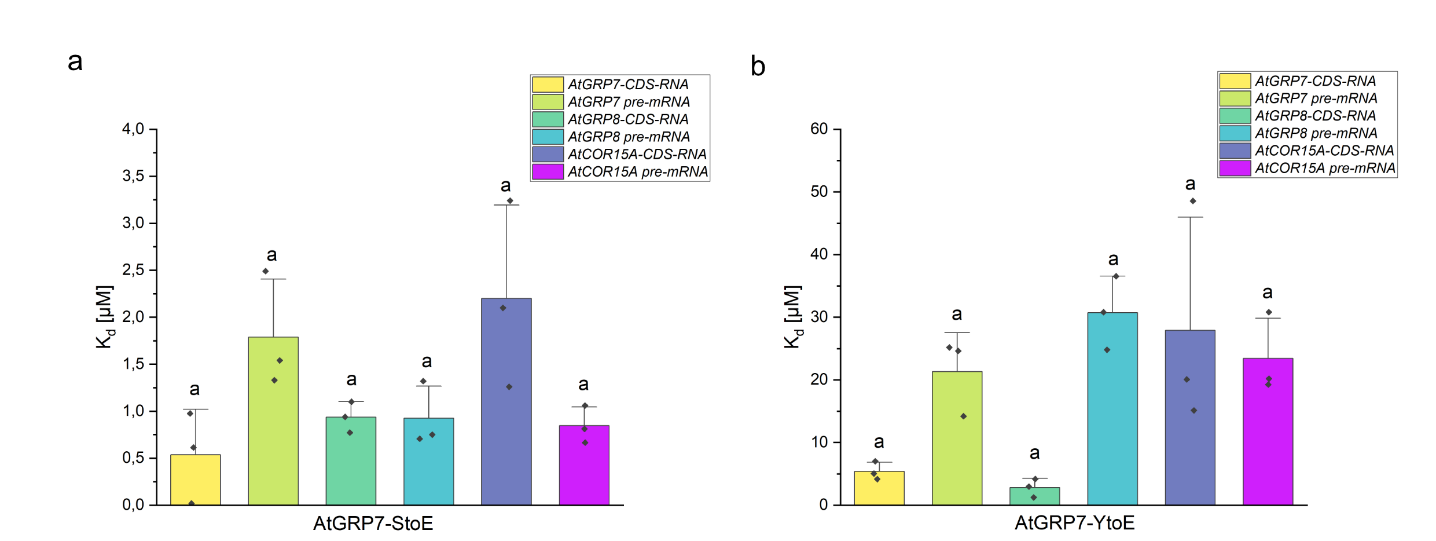


**Supplementary Figure S12: Comparison of dissociation constants (K_d_s) from AtGRP7^StoE^ and AtGRP7^YtoE^ between CDS and pre-mRNA sequences. a)** Boxplot with binding affinities of AtGRP7^StoE^ for CDS and pre-mRNA sequences of different RNAs **b)** Boxplot with binding affinities of AtGRP7^YtoE^ for CDS and pre-mRNA sequences of different RNAs. Y-axis displays the K_d_ in µM (for AtGRP7 and AtGRP7^StoE^ between 0 and 4 µM and for AtGRP7^YtoE^ up to 60 µM) and the X-axis shows the protein measured. The boxes in different colors represent the K_d_ of different RNAs for the protein on the X-axis. The binding affinities were compared with one-way ANOVA and a Tukeys-test for p=0.01. Shared letters indicate no significant difference in binding affinity.


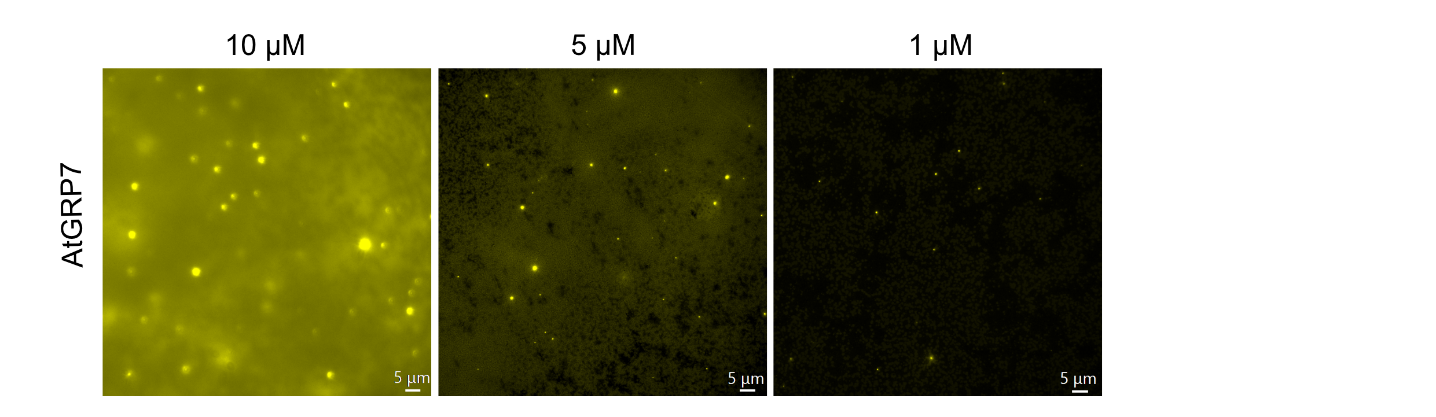


**Supplementary Figure S13:** **Condensation of AtGRP7 at different concentrations.** The condensation of AtGRP7 was investigated at three different concentrations, 10 µM, 5 µM and 1 µM. The white bar represents 5 µm.


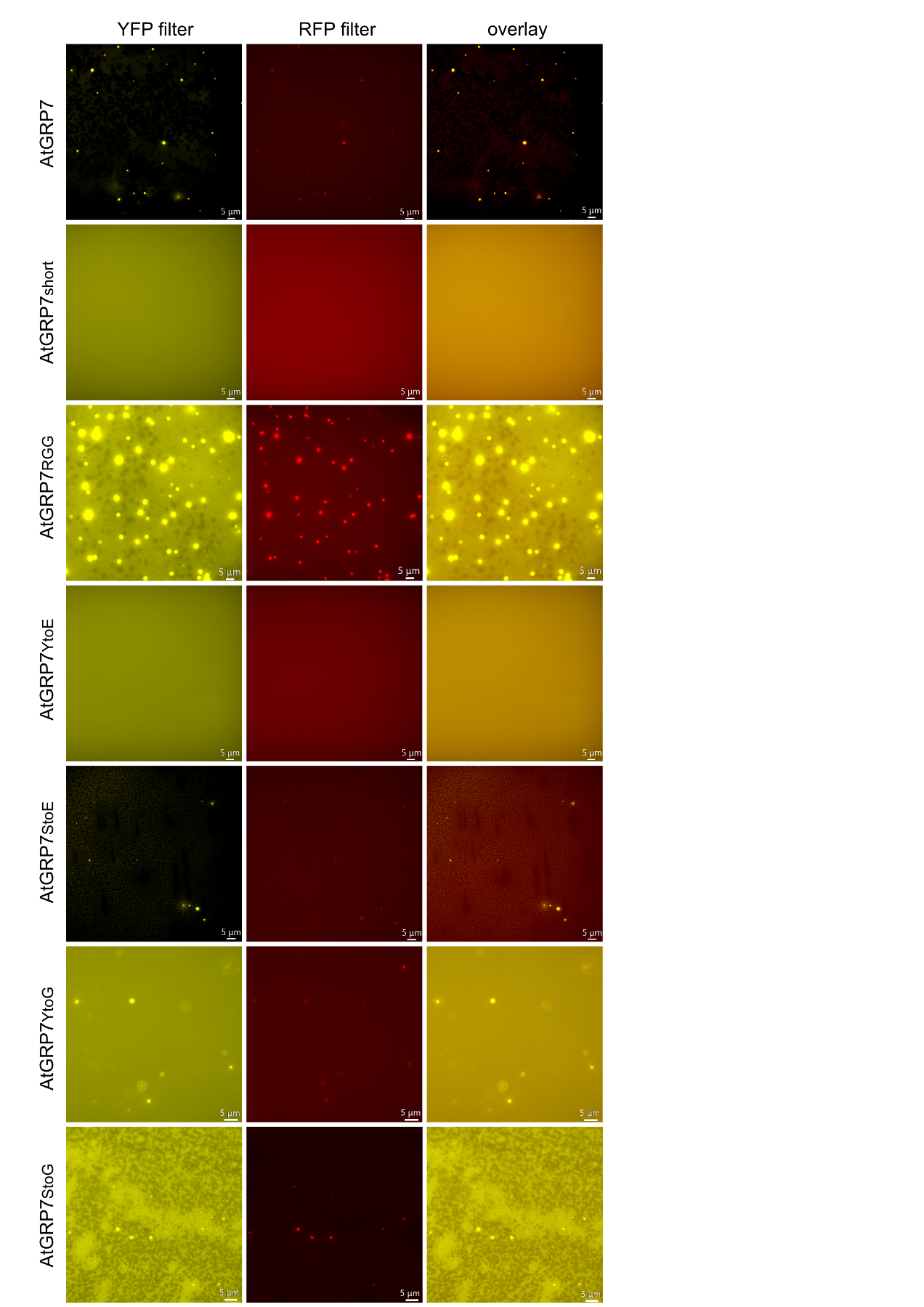


**Supplementary Figure S14: Condensation of AtGRP7 and mutants with 0.5 µM cy3-labeled AtGRP7 CDS RNA.** The proteins had a concentration of 10 µM. The white bar represents 5 µm. Fluorescence was detected with a YFP and an RFP filter.

**Supplementary Table S5: Mean dissociation constant (K_d_) of AtGRP7 for different RNAs.** Mean K_d_ is shown in µM and the standard deviation (SD) is listed next to the K_d_. For each RNA n≥3.

| RNA | Mean K_d_ [µM] | SD [µM] | n |
| --- | --- | --- | --- |
| miR164 | 0.042 | 0.013 | 4 |
| miR164 m6A | 0.142 | 0.017 | 3 |
| miR164 m5C | 0.143 | 0.066 | 3 |
| Bn-miRnovel2 | 0.260 | 0.010 | 3 |
| Bn-miRnovel106 | 0.094 | 0.031 | 3 |
| Bn-miRnovel149 | 0.655 | 0.24 | 3 |
| ds-miR164 | 0.553 | 0.271 | 4 |
| tRNA-met | 0.166 | 0.059 | 4 |
| GRP7-3’UTR | 0.158 | 0.121 | 3 |
| Sc1 RNA NaCl | 6.9 | 4.39 | 3 |
| Sc1 RNA LiCl | 0.463 | 0.001 | 3 |
| GFP-RNA | 9.17 | 3.49 | 3 |
| GFP+GRP7-3’UTR | 25.65 | 5.58 | 3 |
| BnPARCL-CDS RNA | 0.0997 | 0.079 | 4 |
| BnPARCL-CDS RNA w/GA | 0.068 | 0.035 | 3 |
| BnPARCL-CDS RNA m5C | 0.208 | 0.046 | 3 |
| BnPARCL-CDS RNA m6A | 0.024 | 0.005 | 3 |
| ds BnPARCL-CDS RNA | 0.419 | 0.263 | 3 |
| THIOREDOXINH10 | 0.627 | 0.075 | 4 |
| THIOREDOXINH10 m5C | 1.63 | 0.216 | 3 |
| THIOREDOXINH10 RNA m6A | 0.247 | 0.083 | 3 |
| THIOREDOXINH10 RNA w/o GA | 1.91 | 0.164 | 3 |
| CP12-1 RNA | 0.686 | 0.23 | 3 |
| CP12-1 RNA m5C | 0.256 | 0.023 | 3 |
| CP12-1 RNA m6A | 0.429 | 0.094 | 3 |
| BnaC09g46650D RNA | 1.26 | 0.333 | 3 |
| Defensin like protein 3 RNA | 2.72 | 1.26 | 3 |
| Notchless protein RNA | 3.67 | 0.558 | 3 |
| BnGRP7-CDS RNA | 2.97 | 1.26 | 3 |
| BnGRP7+UTR RNA | 0.123 | 0.055 | 3 |
| BnGRP7+UTR+Intron RNA | 0.127 | 0.049 | 3 |
| AtGRP7-CDS RNA | 0.182 | 0.165 | 3 |
| AtGRP7 pre-mRNA | 0.364 | 0.083 | 3 |
| AtGRP8-CDS RNA | 0.177 | 0.086 | 3 |
| AtGRP8 pre-mRNA | 0.156 | 0.083 | 3 |
| AtCOR15A-CDS RNA | 0.062 | 0.022 | 3 |
| AtCOR15A pre-mRNA | 0.224 | 0.162 | 3 |
| siRNA | 0.025 | 0.003 | 3 |

**Supplementary Table S6:** **Mean dissociation constant (K_d_) of BnGRP7 for different RNAs.** Mean K_d_ is shown in µM and the SD is listed next to the K_d_. For each RNA n≥3.

| RNA | Mean K_d_ [µM] | SD [µM] | n |
| --- | --- | --- | --- |
| miR164 | 0.021 | 0.029 | 3 |
| miR164 m6A | 15.85 | 6.22 | 3 |
| miR164 m5C | 16.85 | 6.49 | 3 |
| Bn-miRnovel2 | 0.0058 | 0.005 | 4 |
| Bn-miRnovel106 | 0.042 | 0.035 | 3 |
| Bn-miRnovel149 | 0.004 | 0.003 | 3 |
| GRP7-3’UTR | 0.017 | 0.013 | 3 |
| BnPARCL-CDS RNA | 4.07 | 0.808 | 3 |
| BnPARCL-CDS RNA m5C | 15.7 | 1.47 | 3 |
| BnPARCL-CDS RNA m6A | 7.93 | 2.26 | 3 |
| THIOREDOXINH10 RNA | 3.36 | 1.34 | 3 |
| THIOREDOXINH10 RNA m5C | 12.35 | 1.59 | 3 |
| THIOREDOXINH10 RNA m6A | 9.95 | 7.18 | 3 |
| CP12-1 RNA | 6.08 | 2.47 | 3 |
| CP12-1 RNA m5C | 10.38 | 2.94 | 3 |
| CP12-1 RNA m6A | 13.88 | 5.48 | 3 |
| BnaC05g32740D RNA | 6.69 | 2.00 | 3 |
| BnaC09g46650D RNA | 7.96 | 1.37 | 4 |
| Defensin like protein RNA | 2.78 | 1.28 | 3 |
| Notchless protein RNA | 6.97 | 0.873 | 3 |
| BnaA09g50830D RNA | 2.75 | 1.26 | 3 |
| BnaC09g45930D_neg | 10.05 | 1.41 | 3 |
| BnGRP7-CDS RNA | 7.38 | 2.33 | 4 |
| BnGRP7+UTR RNA | 4.89 | 4.37 | 3 |
| BnGRP7+UTR+Intron RNA | 10.12 | 3.43 | 3 |
| AtGRP7-CDS RNA | 3.72 | 1.63 | 3 |
| AtGRP7 pre-mRNA | 16.23 | 7.66 | 3 |
| AtGRP8-CDS RNA | 9.16 | 1.34 | 3 |
| AtGRP8 pre-mRNA | 0.502 | 0.325 | 3 |
| AtCOR15A-CDS RNA | 0.153 | 0.073 | 3 |
| AtCOR15A pre-mRNA | 0.323 | 0.203 | 3 |

**Supplementary Table S7:** **Mean dissociation constant (K_d_) of AtGRP7^short^ for different RNAs.** Mean K_d_ is shown in µM and the SD is listed next to the K_d_. For each RNA n≥2.

| RNA | Mean K_d_ [µM] | SD [µM] | n |
| --- | --- | --- | --- |
| miR164 | 5.93 | 3.72 | 3 |
| Bn-miRnovel2 | 4.49 | 2.37 | 3 |
| Bn-miRnovel106 | 12.37 | 10.05 | 3 |
| GRP7-3’UTR | 12.18 | 3.86 | 3 |
| BnPARCL-CDS RNA | 4.07 | 0.808 | 3 |
| BnaCNNG53670D RNA | no binding |  | 2 |
| BnGRP7-CDS RNA | no binding |  | 2 |
| BnGRP7+UTR RNA | no binding |  | 2 |
| AtGRP7-CDS RNA | no binding |  | 2 |
| AtGRP7 pre-mRNA | no binding |  | 2 |

**Supplementary Table S8:** **Mean dissociation constant (K_d_) of AtGRP7^YtoE^ for different RNAs.** Mean K_d_ is shown in µM and the SD is listed next to the K_d_. For each RNA n≥3.

| RNA | Mean K_d_ [µM] | SD [µM] | n |
| --- | --- | --- | --- |
| Bn-miRnovel2 | 0.267 | 0.038 | 3 |
| GRP7-3’UTR | 0.701 | 0.178 | 3 |
| AtGRP7-CDS RNA | 5.40 | 1.30 | 3 |
| AtGRP7 pre-mRNA | 21.33 | 6.19 | 3 |
| AtGRP8-CDS RNA | 2.79 | 1.47 | 3 |
| AtGRP8 pre-mRNA | 30.71 | 5.87 | 3 |
| AtCOR15A-CDS RNA | 27.91 | 18.05 | 3 |
| AtCOR15A pre-mRNA | 23.42 | 6.42 | 3 |

**Supplementary Table S9:** **Mean dissociation constant (K_d_) of AtGRP7^YtoG^ for different RNAs.** Mean K_d_ is shown in µM and the SD is listed next to the K_d_. For each RNA n≥3.

| RNA | Mean K_d_ [µM] | SD [µM] | n |
| --- | --- | --- | --- |
| Bn-miRnovel2 | 0.041 | 0.018 | 3 |
| GRP7-3’UTR | 0.356 | 0.083 | 3 |
| AtGRP7-CDS RNA | 7.39 | 2.52 | 3 |
| AtGRP7 pre-mRNA | 19.28 | 9.56 | 3 |
| AtGRP8-CDS RNA | 0.381 | 0.168 | 3 |
| AtGRP8 pre-mRNA | 3.39 | 2.39 | 3 |
| AtCOR15A-CDS RNA | 2.7 | 0.219 | 3 |
| AtCOR15A pre-mRNA | 3.75 | 2.03 | 3 |

**Supplementary Table S10:** **Mean dissociation constant (K_d_) of AtGRP7^StoE^ for different RNAs.** Mean K_d_ is shown in µM and the SD is listed next to the K_d_. For each RNA n≥3.

| RNA | Mean K_d_ [µM] | SD [µM] | n |
| --- | --- | --- | --- |
| Bn-miRnovel2 | 0.029 | 0.004 | 3 |
| GRP7-3’UTR | 0.072 | 0.015 | 3 |
| AtGRP7-CDS RNA | 0.536 | 0.484 | 3 |
| AtGRP7 pre-mRNA | 1.79 | 0.618 | 3 |
| AtGRP8-CDS RNA | 0.937 | 0.164 | 3 |
| AtGRP8 pre-mRNA | 0.923 | 0.343 | 3 |
| AtCOR15A-CDS RNA | 2.47 | 0.664 | 3 |
| AtCOR15A pre-mRNA | 0.738 | 0.103 | 3 |

**Supplementary Table S11:** **Mean dissociation constant (K_d_) of AtGRP7^StoG^ for different RNAs.** Mean K_d_ is shown in µM and the SD is listed next to the K_d_. For each RNA n≥3.

| RNA | Mean K_d_ [µM] | SD [µM] | n |
| --- | --- | --- | --- |
| Bn-miRnovel2 | 0.022 | 0.006 | 3 |
| GRP7-3’UTR | 0.052 | 0.011 | 3 |
| AtGRP7-CDS RNA | 1.17 | 0.941 | 3 |
| AtGRP7 pre-mRNA | 0.606 | 0.601 | 4 |
| AtGRP8-CDS RNA | 0.493 | 0.186 | 4 |
| AtGRP8 pre-mRNA | 1.37 | 0.487 | 3 |
| AtCOR15A-CDS RNA | 2.3 | 1.63 | 4 |
| AtCOR15A pre-mRNA | 2.23 | 1.06 | 3 |

**Supplementary Table S12:** **Mean dissociation constant (K_d_) of AtBnGRP7 for different RNAs.** Mean K_d_ is shown in µM and the SD is listed next to the K_d_. For each RNA n≥3.

| RNA | Mean K_d_ [µM] | SD [µM] | n |
| --- | --- | --- | --- |
| Bn-miRnovel2 | 0.035 | 0.045 | 3 |
| GRP7-3’UTR | 0.018 | 0.005 | 3 |
| BnGRP7-CDS RNA | 0.414 | 0.115 | 3 |
| BnGRP7 UTR+Intron | 0.158 | 0.038 | 3 |
| AtGRP7-CDS RNA | 0.107 | 0.045 | 3 |
| AtGRP7 pre-mRNA | 0.172 | 0.103 | 3 |
| AtGRP8-CDS RNA | 0.135 | 0.041 | 3 |
| AtGRP8 pre-mRNA | 0.312 | 0.135 | 3 |
| AtCOR15A-CDS RNA | 0.548 | 0.279 | 3 |
| AtCOR15A pre-mRNA | 0.741 | 0.153 | 3 |

**Supplementary Table S13: Mean dissociation constant (K_d_) of AtGRP7^RGG^ for different RNAs.** Mean K_d_ is shown in µM and the SD is listed next to the K_d_. For each RNA n≥3.

| RNA | Mean K_d_ [µM] | SD [µM] | n |
| --- | --- | --- | --- |
| GRP7-3’UTR | 0.031 | 0.013 | 3 |
| AtGRP7-CDS RNA | 1.52 | 1.06 | 3 |

**Supplementary Methods**

***In silico* motif analysis of sequenced and MST-measured long RNAs**

Using bedtools-getfasta (v2.30.0)^[1]^ on Galaxy Europe (Bjoern A. Gruening (2014), Galaxy wrapper), all sequences of BnGRP7 enriched phloem RNAs were extracted into one fasta file. This fasta file was used in MEME (Multiple Em for Motif Elicitation) to identify common sequence motifs in phloem RNAs enriched by GRP7. Furthermore, sequences of MST-tested, long RNAs were analyzed for enriched motifs using MEME-suite v5.5.5 (https://meme-suite.org/meme/tools/meme)^[2]^. Both times, the default settings were used.

***In silico* structural analysis of RNAs**

For the *in silico* structural analysis of RNAs, RNAfold v2.6.3 (http://rna.tbi.univie.ac.at/cgi-bin/RNAWebSuite/RNAfold.cgi )^[3,4]^ was used with default settings.

***In silico* structural analysis and comparison of AtGRP7 and its mutants**

AlphaFold2 v2.3.1^[5]^ was used with standard settings at Galaxy Europe (https://usegalaxy.eu/root?tool_id=toolshed.g2.bx.psu.edu/repos/galaxy-australia/alphafold2/alphafold/2.3.1+galaxy5) to determine the structures of AtGRP7, AtGRP7^short^, AtGRP7^StoE^, AtGRP7^YtoE^, AtGRP7^StoG^, AtGRP7^YtoG^, BnGRP7, and AtBnGRP7. The structures were then compared with the structure alignment tool of RCSB PDB (https://www.rcsb.org/alignment) using the jFATCAT-flexible v2.0 algorithm^[6,7]^

**Supplementary References**

1. Quinlan, A. R. & Hall, I. M. BEDTools: a flexible suite of utilities for comparing genomic features. *Bioinformatics* **26**, 841–842 (2010).

2. Bailey, T. L., Johnson, J., Grant, C. E. & Noble, W. S. The MEME Suite. *Nucleic Acids Res.* **43**, W39–W49 (2015).

3. Gruber, A. R., Lorenz, R., Bernhart, S. H., Neuböck, R. & Hofacker, I. L. The Vienna RNA Websuite. *Nucleic Acids Res.* **36**, W70–W74 (2008).

4. Lorenz, R. *et al.* ViennaRNA Package 2.0. *Algorithms Mol. Biol.* **6**, 1–14 (2011).

5. Jumper, J. *et al.* Highly accurate protein structure prediction with AlphaFold. *Nature* **596**, 583–589 (2021).

6. Ye, Y. & Godzik, A. Flexible structure alignment by chaining aligned fragment pairs allowing twists. *Bioinformatics* **19**, ii246–ii255 (2003).

7. Li, Z., Jaroszewski, L., Iyer, M., Sedova, M. & Godzik, A. FATCAT 2.0: towards a better understanding of the structural diversity of proteins. *Nucleic Acids Res.* **48**, W60–W64 (2020).
